# SH3BP5L triggers the RAB11A-regulated integrin recycling network implicated in breast cancer metastasis

**DOI:** 10.1172/JCI192705

**Published:** 2026-02-02

**Authors:** Huayi Li, Maria Chiara De Santis, Francesco A. Tucci, Daniela Tosoni, Ping Zhang, Meredith L. Jenkins, Giulia Villari, Maria Grazia Filippone, Elisa Guerrera, Simone Tealdi, Luca Gozzelino, Federico Gulluni, Lorenzo Prever, Cristina Zanini, Marco Forni, Irene Franco, Miriam Martini, John E. Burke, Guido Serini, Carlo Cosimo Campa, Salvatore Pece, Jean Piero Margaria, Emilio Hirsch

**Affiliations:** 1 Department of Molecular Biotechnology and Health Sciences, Molecular Biotechnology Center “G. Tarone,” University of Torino, Torino, Italy.; 2 IEO, European Institute of Oncology IRCCS, Milan, Italy.; 3 Department of Oncology and Hemato-Oncology, University of Milano, Milano, Italy.; 4 Department of Biochemistry and Microbiology, University of Victoria, Victoria, British Columbia, Canada.; 5 Department of Oncology, University of Torino Medical School, Candiolo, Torino, Italy.; 6 Candiolo Cancer Institute, Fondazione del Piemonte per l’Oncologia (FPO) – IRCCS, Candiolo, Torino, Italy.; 7 Department of Mechanical and Aerospace Engineering, Politecnico di Torino, Torino, Italy.; 8 Italian Institute for Genomic Medicine, Candiolo, Torino, Italy.; 9 Università Vita-Salute San Raffaele Milan, Italy.; 10 Department of Biochemistry and Molecular Biology, The University of British Columbia, Vancouver, British Columbia, Canada.; 11 Somatic mutation mechanisms Unit, Division of Genetics and Cellular Biology, Ospedale San Raffaele – IRCCS, Milan, Italy.

**Keywords:** Cell biology, Oncology, Breast cancer, Cell migration/adhesion, Protein traffic

## Abstract

Metastatic progression in aggressive breast cancer (BC) depends on a tightly controlled vesicular recycling network regulated by RAB11, a small guanosine triphosphate enzyme (GTPase). In a cohort of more than 1,000 patients with BC, we identified SH3BP5L as the most highly expressed guanine nucleotide exchange factor (GEF) for RAB11A. High SH3BP5L expression marked an advanced tumor stage, distant metastasis, and poor prognosis, with significant associations in human epidermal growth factor receptor 2–positive (HER2^^+^^) and triple-negative breast cancer (TNBC). Using Förster resonance energy transfer (FRET) sensors and artificial intelligence– (AI-assisted) microscopy, we showed that cargo delivery to the plasma membrane required SH3BP5L-dependent activation of RAB11A and assembly of a complex with the anterograde motor KIF5B. This trafficking governed key metastatic features of TNBC, including β1 integrin recycling and α3β1 integrin surface exposure. Inhibition of SH3BP5L or its GEF activity reduced cell spreading in zebrafish and lung metastasis in mouse models, revealing a previously unidentified driver of BC dissemination and a potential therapeutic vulnerability.

## Abstract 2

In triple negative breast cancers, we identified the RAB11A GEF SH3BP5L as a crucial target to lower lung metastasis and cell spreading.

## Introduction

Triple-negative breast cancer (TNBC) is an aggressive subtype of breast cancer (BC) with limited therapeutic options and a poor prognosis. A defining feature of TNBC is its high propensity for metastasis, particularly to the brain, liver, and lungs, which significantly contributes to mortality (1). This metastatic behavior depends on complex molecular and cellular processes, many of which are controlled by vesicular trafficking systems regulated by RAB guanosine triphosphate enzymes (GTPases). These molecular switches coordinate endocytosis, recycling, and exocytosis, thereby sustaining adhesion, polarity, and motility (2, 3).

Within the RAB family, RAB11 isoforms, including RAB11A, RAB11B, and RAB25, are central regulators of the recycling pathway, which in turn influences cancer progression and metastatic dissemination (3–6). Like other RABs, RAB11 cycles between an inactive guanosine diphosphate–bound (GDP-bound) and an active GTP-bound state. This transition is tightly regulated by 2 classes of proteins: GTPase-activating proteins (GAPs), which promote inactivation, and guanine nucleotide exchange factors (GEFs), which mediate activation. GAPs such as TBC1D9B ensure timely GTP hydrolysis and restrict inappropriate signaling (7, 8). On the activating side, RAB11 can be stimulated by the multisubunit TRAPPII complex (9–11), which promotes GDP-GTP exchange through a composite catalytic surface. Conversely, SH3BP5 and SH3BP5L have been identified as the first “standalone” RAB11 GEFs, unique in directly engaging the switch I and II regions of RAB11 to catalyze nucleotide exchange (12, 13). Both proteins are further controlled by tankyrase, a poly-ADP-ribose polymerase that modifies distinct sites on each paralog, thereby suppressing their GEF activity and reducing RAB11A-GTP levels (14). Despite these similarities, the 2 paralogs diverge significantly in both sequence and function. SH3BP5L contains an extended N-terminal region (12, 13) and shows substantially higher catalytic efficiency toward RAB11A (~8.1 × 10^4^ vs. ~3.5 × 10^4^ M × s) (13). SH3BP5, originally described as Sab, has been implicated instead in negative regulation of Bruton’s tyrosine kinase (15) and in sustaining JNK activation under stress (16–18). In contrast, SH3BP5L has emerged as the paralog primarily dedicated to polarized trafficking (13, 14), positioning it as a strong candidate to regulate RAB11-dependent recycling of integrins and other surface effectors whose delivery to the plasma membrane sustains directed migration.

Indeed, activation of RAB11is essential for the spatiotemporal coordination of vesicle transport toward the cell surface (4). Active RAB11 interacts with effectors such as RAB11-FIP3, EXOC6, DLIC, and Myo5b to mediate vesicle tagging, docking, and fusion, ensuring delivery from the endocytic recycling compartment (ERC) to the plasma membrane (19–22). In addition, RAB11 cooperates with kinesins such as KIF13A to promote anterograde transport of adhesion receptors to lamellipodia and filopodia, thereby supporting cell motility (4, 23). Through these mechanisms, RAB11 governs the recycling of internalized cargos back to the plasma membrane, a process that is essential for adhesion, migration, and invasion (3, 5, 6, 24). Although RAB11 overexpression has been linked to metastasis (25), the upstream GEFs and GAPs that control RAB11-dependent integrin recycling in invasive cancers have remained undefined.

In this study, we demonstrate that SH3BP5L was overexpressed in a large cohort of patients with BC, with the highest levels observed in patients with TNBC. In TNBC cell lines, SH3BP5L activates RAB11A and promotes its interaction with the kinesin KIF5B, thereby directing recycling of active β1 integrins and surface exposure of α3β1 at the plasma membrane. This pathway sustains TNBC migration and metastasis, establishing SH3BP5L as the first Rab11 GEF, to our knowledge, to be functionally linked to BC progression and highlighting it as a promising therapeutic target.

## Results

### SH3BP5L overexpression is associated with metastasis of aggressive BCs.

**Figure 1:**
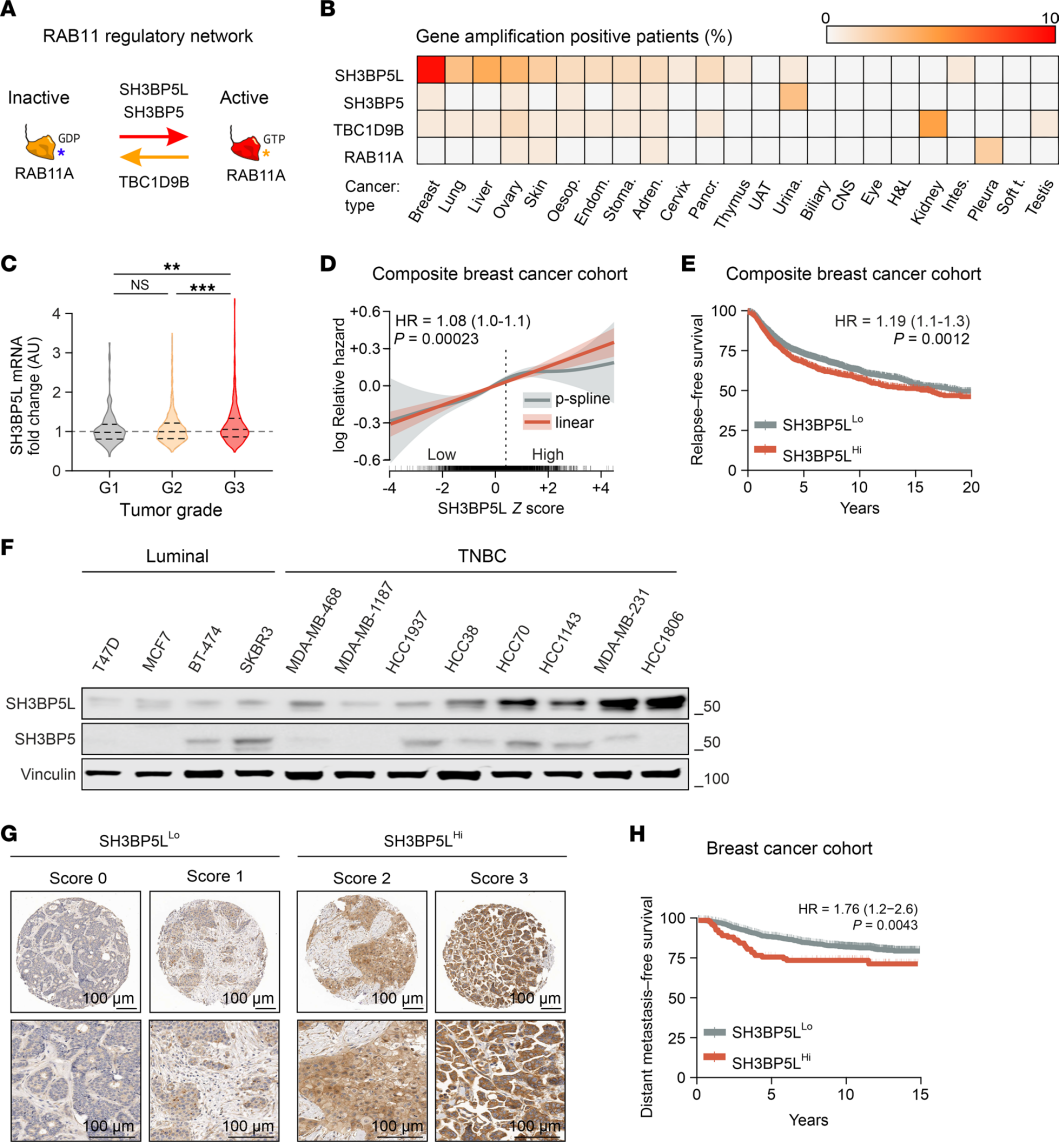
SH3BP5L expression in BC and association with patient outcomes. (**A**) Schematic representation of the RAB11 regulatory network. RAB11-GDP is activated by its GEFs, SH3BP5L and SH3BP5, and inactivated by its GAP, TBC1D9B. (**B**) Copy number alterations of SH3BP5L, SH3BP5, TBC1D9B, and RAB11A across cancer types in TCGA database. Adren., adrenal gland; Oesop., esophagus; Endom., endometrium; Stoma., stomach; Pancr., pancreas; UAT, upper aerodigestive tract; Urina., urinary tract; H&L, hematopoietic and lymphoid; Intes., large intestinal; Soft t., soft tissue. (**C**) Violin plot showing *SH3BP5L* mRNA expression across tumor grades in the METABRIC dataset (*n* = 1,893). **P = 0.002 and ***P < 0.001, by Wilcoxon–Mann-Whitney *U* test. (**D** and **E**) Association of SH3BP5L expression with RFS in a composite BC cohort. (**D**) Continuous analysis of the log relative hazard against SH3BP5L expression. Both a linear fit (red) and a penalized spline fit (gray) are shown. The HR and log-rank test *P* value refer to the linear term. Shaded areas represent 95% CIs. Dashed line indicates the upper tertile cutoff. (**E**) Kaplan-Meier analysis of patients stratified according to SH3BP5L^hi^ (upper tertile) and SH3BP5L^lo^ expression. *P* denotes the log-rank *P* value; *n* = 4,005. (**F**) Representative immunoblots of SH3BP5L and SH3BP5 expression normalized to vinculin in luminal and TNBC cell lines. (**G**) Immunohistochemical assessment of SH3BP5L expression in patients with BC (*n* = 1590). Representative images of SH3BP5L^lo^ tumors (*n* = 1,470, score 0/1) and SH3BP5L^hi^ tumors (*n* = 120, score 2/3). Scale bars: 100 μm. (**H**) DMFS analysis of patients with SH3BP5L^hi^ versus SH3BP5L^lo^ BC. *P* denotes the log-rank test *P* value; *n* = 1,590.

To explore the contribution of the RAB11A-regulated vesicular recycling network in human cancers (Figure 1A), we analyzed copy number alterations (CNAs) of RAB11A, its GEFs (SH3BP5, SH3BP5L), and its GAP (TBC1D9B) across malignancies in The Cancer Genome Atlas (TCGA) database. Multiple cancer types displayed amplification of SH3BP5L, with the highest frequency (10%) in BC (Figure 1B). We therefore investigated the implications of SH3BP5L expression in this context.

Transcriptomics analyses of public BC datasets showed that *SH3BP5L* mRNA levels were higher in high-grade tumors (Figure 1C) and enriched in TNBC compared with luminal tumors (Supplemental Figure 1, A and B; supplemental material available online with this article; https://doi.org/10.1172/JCI192705DS1) (26, 27). In METABRIC (26), SH3BP5L was also elevated in human epidermal growth factor receptor 2–positive (HER2^+^) tumors, with no significant difference between TNBC and HER2^+^ BC (Supplemental Figure 1B). To assess the clinical significance, we analyzed a Composite Breast Cancer cohort integrating the METABRIC and KMplotter microarray datasets to increase statistical power and avoid single-cohort biases. Both cohorts have long-term follow-up (median of 13.0 and 6.9 years for METABRIC and KMplotter, respectively) and showed a similar distribution of PAM50 subtypes (Supplemental Figure 1C). Across these datasets, *SH3BP5L* mRNA levels were associated with shorter relapse-free survival (RFS), with increasing expression corresponding to a monotonic, approximately linear rise in relative hazard in the aggregated cohort (Figure 1D) and in each dataset considered separately, with similar effect size and statistical significance (Supplemental Figure 1, D and E). Consistently, when patients were dichotomized, RFS was shorter for patients with SH3BP5L^hi^ tumors than for those with SH3BP5L^lo^ tumors (Figure 1E).

By contrast, the close paralog SH3BP5 showed no enrichment in any molecular subtype (Supplemental Figure 1F and Supplemental Methods) and was instead associated with a favorable prognosis (Supplemental Figure 1, G and H). Protein analyses confirmed this divergence: TNBC cell lines expressed significantly more SH3BP5L than did luminal cell lines, with the highest levels detected in metastatic HCC1806 and MDA-MB-231 cells, whereas SH3BP5 showed no consistent enrichment (Figure 1F and Supplemental Figure 1I).

To confirm the clinical relevance, we assessed SH3BP5L protein expression in a retrospective BC cohort of 2,453 patients with complete clinicopathological annotation and long-term follow-up (median 14.5 years) (28, 29). A total of 1,590 viable tumor cores were scored for overall SH3BP5L staining intensity by IHC. On the basis of this evaluation, most patients (*n* = 1,470) were classified as SH3BP5L^lo^, whereas 120 were SH3BP5L^hi^ (Figure 1G and Table 1). SH3BP5L^hi^ status was associated with indicators of aggressive disease, including higher Ki67 levels, grade, and pathological tumor stage (Table 1). Clinically, SH3BP5L^hi^ patients showed significantly reduced distant metastasis–free survival (DMFS) (Figure 1H).

We then analyzed SH3BP5L expression across BC molecular subtypes. SH3BP5L^hi^ tumors were enriched in TNBC (16%) compared with SH3BP5L^lo^ tumors (8.9%) or the cohort average (9.4%), and also included a higher proportion of HER2^+^ cases (28%) (Table 1). Patients with SH3BP5L^hi^ cancer had largely invasive ductal/no special type and showed no association with rarer histological variants (Table 1). Subtype-specific survival analyses revealed that high SH3BP5L expression was significantly associated with reduced DMFS in HER2^+^ cancers. A similar trend was observed in TNBC, although statistical significance was likely limited by the smaller number of TNBC cases (Supplemental Figure 2A). By contrast, we observed no prognostic association in luminal tumors.

In agreement, subgroup analysis in the Composite Breast Cancer cohort confirmed SH3BP5L risk stratification in HER2^+^ and basal-like subtypes (Supplemental Figure 2B). Conversely, SH3BP5 did not consistently stratify HER2^+^ and was positively associated with a good prognosis in luminal and basal-like disease (Supplemental Figure 2C).

Since HER2^+^ cancers already benefit from effective targeted therapies, we concentrated subsequent analyses on TNBC, for which therapeutic options remain limited and new approaches are urgently needed to reduce metastatic burden.

### The GEF activity of SH3BP5L promotes TNBC cell migration via RAB11A activation.

**Figure 2:**
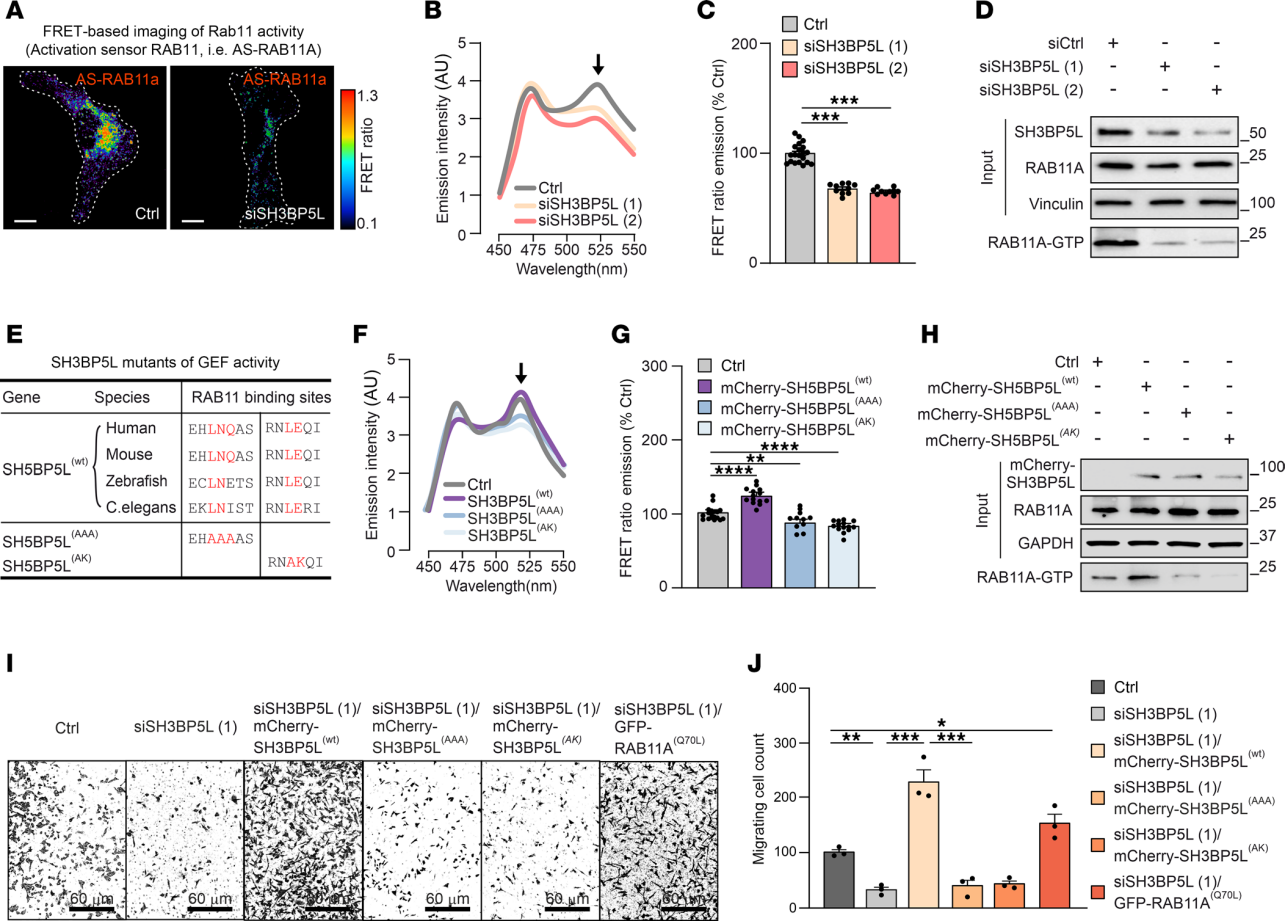
SH3BP5L affects RAB11A activation. (**A**) Representative pseudocolored confocal image of the FRET ratio in MDA-MB-231 cells transfected with SH3BP5L-silenced AS-RAB11A. Scale bars: 5 μm. (**B**) Spectrophotometry assay quantification of FRET ratio emission intensity in lysate obtained from HEK293T cells transfected with AS-RAB11A FRET sensor and SH3BP5L siRNAs. *n* ≥6 independent experiments. (**C**) Quantification of the FRET ratio from confocal image analysis of MDA-MB-231 cells transfected with AS-RAB11A sensor and SH3BP5L siRNAs. *n* ≥9 cells. (**D**) Representative Western blot of endogenous RAB11-GTP content measured with a RAB11 activation pull-down assay using GST-RAB11FIP3 (specifically binds to the active GTP loaded form of RAB11A) in SH3BP5L-depleted MDA-MB-231 cells. (**E**) Table representing SH3BP5L conserved regions for the binding of RAB11A and the overlapping putative SH3BP5L amino acid residues. (**F**) Spectrophotometry assay quantification of FRET ratio emission intensity in lysate obtained from HEK293T cells transfected with AS-RAB11A FRET sensor and mCherry-SH3BP5L^(WT)^, mCherry-SH3BP5L^(AAA)^, or mCherry-SH3BP5L^(AK)^ constructs. *n* ≥7 independent experiments. (**G**) Quantification of the FRET ratio from confocal image analysis of MDA-MB-231 cells transfected with AS-RAB11A sensor and mCherry-SH3BP5L^(WT)^, mCherry-SH3BP5L^(AAA)^, or mCherry-SH3BP5L^(AK)^ constructs. *n* ≥10 cells. (**H**) Representative Western blot of endogenous RAB11-GTP content measured with a RAB11 activation pull-down assay using GST-RAB11FIP3 (specifically binds to the active GTP-loaded form of RAB11A) in MDA-MB-231 cells transfected with mCherry (Ctrl), mCherry-SH3BP5L^(WT)^, mCherry-SH3BP5L^(AAA)^, or mCherry-SH3BP5L^(AK)^ constructs. (**I** and **J**) Representative crystal violet staining (**I**) and relative quantification (**J**) of a Transwell assay of MDA-MB-231 cells depleted of SH3BP5L (siSH3BP5L) and transfected with mCherry-SH3BP5L^(WT)^, mCherry-SH3BP5L^(AAA)^, mCherry-SH3BP5L^(AK)^, or constitutively active GFP-RAB11A^(Q70L)^ constructs. Transfection efficiency was confirmed separately, with at least 70% of cells fluorescently labeled. Scale bars: 60 μm (**I**). Data represent the mean of at least 3 independent experiments ± SEM. *P < 0.05, **P < 0.01, and ***P < 0.005, by 1-way ANOVA. Ctrl, control.

We investigated the role of SH3BP5L in aggressive BC by knockdown (KD) of SH3BP5L in MDA-MB-231 cells, a cellular model of TNBC with high SH3BP5L expression. A previously developed Förster resonance energy transfer (FRET) sensor to measure RAB11A activation (AS-RAB11A) (30, 31) highlighted a decrease in RAB11A activation upon SH3BP5L KD (siSH3BP5L) (Figure 2, A–C, and Supplemental Figure 3A). This finding was independently validated using a pull-down assay with the recombinant RAB11-GTP–interacting domain of FIP3 as a probe (30, 32). This second experiment confirmed reduced RAB11A activity with decreased SH3BP5L expression (Figure 2D, Supplemental Figure 3B, and Supplemental Methods). In contrast, overexpression of WT SH3BP5L restored and enhanced RAB11A activity, as measured by the FRET sensor (Supplemental Figure 3, C and D).

Next, we assessed the GEF activity of SH3BP5L. We introduced point mutations into conserved residues of the SH3BP5L GEF domain, previously shown to abolish SH3BP5 GEF activity (13), thereby generating the SH3BP5L^(AAA)^ and SH3BP5L^(AK)^ variants (Figure 2E). Measurements of RAB11A activity by spectrofluorimetric analysis, confocal microscopy, and FIP3 pull-down assays showed that SH3BP5L^(AAA)^ and SH3BP5L^(AK)^ induced a 50% reduction in RAB11A activation compared with SH3BP5L^(WT)^ (Figure 2, F–H, Supplemental Figure 3E, and Supplemental Methods).

Given the involvement of SH3BP5L in BC aggressiveness (Figure 1), we hypothesized that the SH3BP5L/RAB11A axis could positively influence metastatic spreading. We induced overexpression of RAB11A^(WT)^ and the constitutively active RAB11A^(Q70L)^ in the TNBC cell line MDA-MB-231. Both transfections increased migration, whereas the inactive mutant RAB11A^(S25N)^ had the opposite effect, i.e., a reduction in migration (Supplemental Figure 3, F and G). We also observed a significant reduction in migration in SH3BP5L-KD cells (siSH3BP5L) (Figure 2, I and J). Remarkably, this effect appeared specific to cell movement, as cell proliferation remained unaffected in siSH3BP5L cells (Supplemental Figure 3H). The reduction in migration was fully rescued by reintroducing WT SH3BP5L, whereas GEF-deficient mutants (AAA and AK) failed to restore the impaired migration in siSH3BP5L cells (Figure 2, I and J). Similarly, the reduced migration observed in siSH3BP5L cells was reversed by genetic activation of the cells’ downstream effector RAB11A, achieved by transfection of the constitutively active RAB11A^(Q70L)^ mutant (Figure 2, I and J). This rescue did not change when we induced concomitantly expression of RAB11A^Q70L^ and either one of the GEF-deficient SH3BP5L mutants in siSH3BP5L cells (Supplemental Figure 3, F and G). Notably, the overexpression of SH3BP5L, but not of its closely related homolog SH3BP5, resulted in migratory improvements (Supplemental Figure 3, I and J), pointing to a specific requirement of the GEF activity of SH3BP5L for TNBC cell migration.

Altogether, these results establish SH3BP5L as a key regulator of the migratory capacity of MDA-MB-231 cells, and this function was strictly mediated by SH3BP5L GEF activity toward RAB11A.

### SH3BP5L-activated RAB11A interacts with KIF5B to direct vesicles to the plasma membrane.

**Figure 3:**
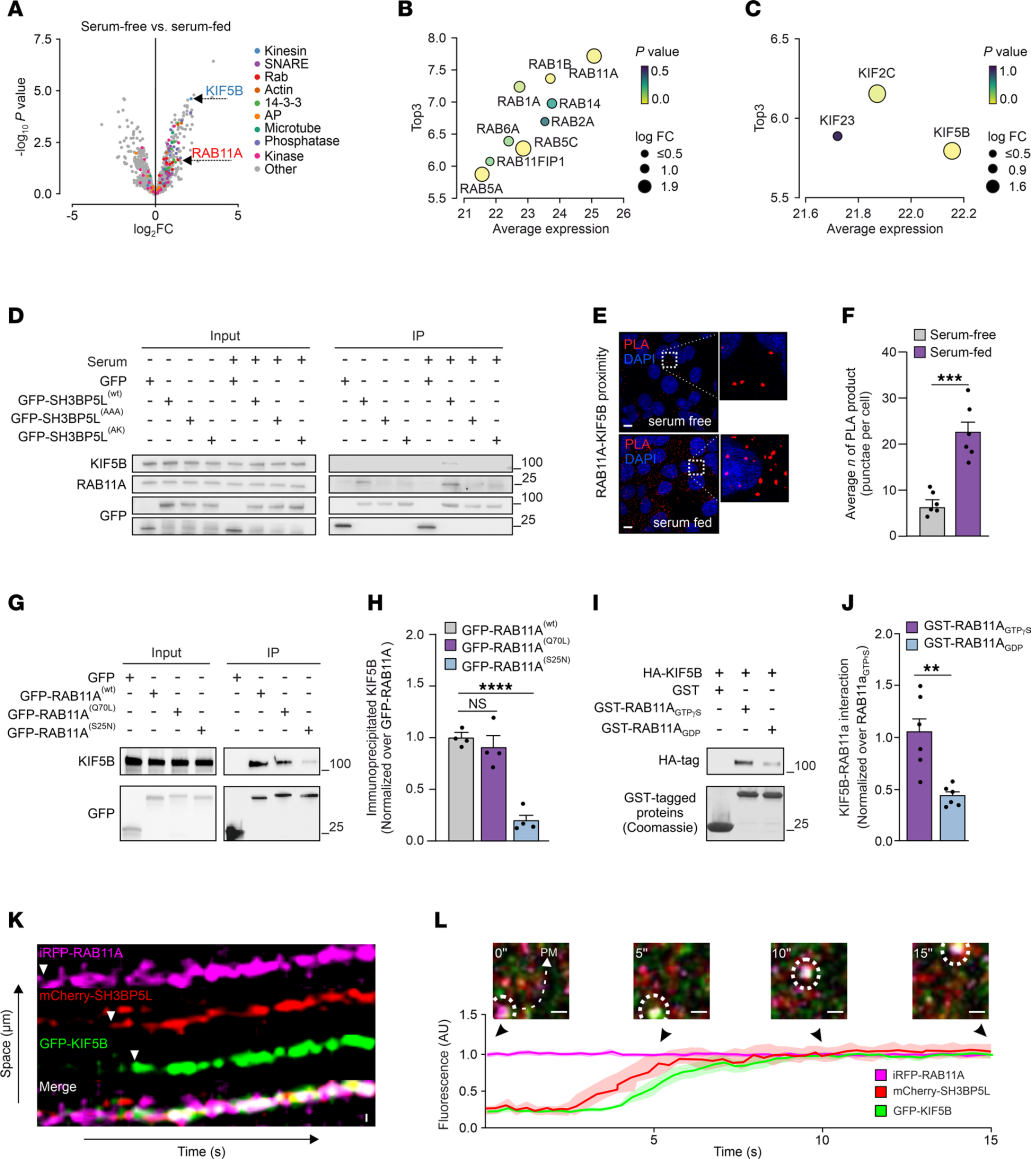
SH3BP5L interacts with KIF5B to recycle cargo back to the plasma membrane. (**A**) Volcano plot of mass spectrometry analysis performed from GFP immunoprecipitation of HEK293T cells transfected with GFP or GFP-SH3BP5L in the control (serum-free) or recycling (serum-fed) condition. (**B** and **C**) Bubble plot illustrating the relationship between the average expression and Top3 metric for (**B**) RAB and (**C**) kinesin family proteins. Bubble size represents the log fold change (log FC), and bubble color corresponds to the *P* value. (**D**) Representative Western blot of GFP immunoprecipitation performed in HEK293T cells transfected with GFP, GFP-SH3BP5L^(WT)^, GFP-SH3BP5L^(AAA)^ , or GFP-SH3BP5L^(AK)^ constructs in serum-starved or -fed conditions. (**E** and **F**) Representative confocal images (**E**) and quantification (**F**) of a PLA for RAB11A and KIF5B in MDA-MB-231 cells. *n* = 6 images for each group from 3 independent experiments. Scale bars: 5 μm. (**G** and **H**) Representative Western blots (**G**) and relative quantification (**H**) of GFP immunoprecipitation performed on HEK293T cells transfected with GFP, GFP-RAB11A^(WT)^, GFP-RAB11A^Q70L^, or GFP-RAB11A^(S25N)^ constructs. (**I** and **J**) Representative Western blots (**I**) and relative quantification (**J**) of a pull-down assay for GST-RAB11A loaded with GDP or GTPγS performed on HEK293T cells transfected with HA-KIF5B. (**K**) Kymograph of fluorescence intensity for mCherry-SH3BP5L (red) and GFP-KIF5B (green) along an iRFP-RAB11A-labeled (magenta) vesicle over time. Merge shows the colocalization dynamics. (**L**) Representative image of the vesicle positive for iRFP-RAB11A, mCherry-SH3BP5L, and GFP-KIF5B (top) showing its transport toward the plasma membrane (PM). Quantification of relative fluorescence intensity over time for iRFP-RAB11A, mCherry-SH3BP5L, and GFP-KIF5B, with shaded regions indicating standard deviation (bottom). Scale bars: 1 μm. Data represent the mean of at least 3 independent experiments ± SEM. **P < 0.01, ***P < 0.005, and ****P < 0.001, by 1-way ANOVA.

By activating RAB11A in TNBC cells, SH3BP5L may promote the recycling of cargo vesicles. This process is enabled by various molecular players that facilitate vesicle movement and positioning. In particular, serum stimulation can trigger cargo vesicles to recycle back to the plasma membrane and facilitate cell migration by providing multiple cues, including growth factors and extracellular matrix (ECM) components. To pinpoint specific molecular actors that interact with SH3BP5L and contribute to its GEF activity–dependent role in metastasis, we performed proteomics analysis of SH3BP5L-GFP pull-down assays. To this aim, we conducted a comparative analysis of the protein complexes immunoprecipitated with SH3BP5L in 2 distinct cellular scenarios: (a) a control condition, represented by cells maintained under serum starvation (serum-free) and (b) a test condition, represented by cells subjected to serum starvation followed by a 40-minute reexposure to an FBS treatment (serum-fed) leading to plasma membrane recycling of cargo proteins (32–35) (Figure 3A). This approach revealed substantial enrichment of vesicular transport proteins in the serum-treated SH3BP5L sample (Supplemental Figure 4A).

Through this procedure, we identified elements of the SH3BP5L interactome, from which we selected 92 proteins associated with intracellular vesicular traffic, including Rabs, kinesins, as well as microtubule components (Figure 3A). Statistical analysis highlighted RAB11A as the most enriched candidate among all the Rabs captured by our assay (Figure 3B), thus confirming the pivotal role of RAB11A as the primary target modulated by SH3BP5L. Particularly striking was the augmented interaction of the complex with the kinesin family member 5B (KIF5B) (Figure 3C).

Next, we validated the interaction between the SH3BP5L complex and KIF5B using immunoprecipitation. We found that the interactions between SH3BP5L, RAB11A, and KIF5B were significantly enhanced following serum stimulation and that these interactions were blocked by SH3BP5L-inactivating mutations (Figure 3D, Supplemental Figure 4, B and C, and Supplemental Methods). The serum-induced association between RAB11A and KIF5B was further confirmed through proximity ligation assays (PLAs). The assay demonstrated that these 2 proteins were assembled in a common complex (Figure 3, E and F, Supplemental Figure 4, D and E, and Supplemental Methods). Conversely, PLA analysis revealed negligible interactions between KIF5B and SH3BP5, the homolog of SH3BP5L (Supplemental Figure 4, F and G). Collectively, these findings support the assumption that SH3BP5L, rather than SH3BP5, activates a pool of RAB11A, which can recruit KIF5B, potentially coordinating the anterograde transport of metastasis-promoting cargo vesicles.

To further validate this model, we examined the interplay between RAB11A and KIF5B through anti-GFP immunoprecipitation and a PLA assay. Consistent with our hypothesis, both assays confirmed that KIF5B interacts with both WT GFP-RAB11A and its constitutively active form (Q70L), but not with the dominant-negative form (S25N) (Figure 3, G and H, Supplemental Figure 4, F and G, and Supplemental Methods). In vitro GST pull-down assays provided additional support, showing that KIF5B preferentially interacted with RAB11A in its GTP-bound form (GST-RAB11A_GTPγS_) over the GDP-bound form (GST-RAB11A_GDP_) (Figure 3, I and J, and Supplemental Methods). Analysis of the interaction between GTP-loaded RAB11A and a KIF5B dimer by AlphaFold3 (36) showed a potential direct interaction between these proteins at the C-terminal end of KIF5B (Supplemental Figure 4H), with preferential affinity for GTP- versus GDP-loaded RAB11 (Supplemental Figure 4I).

Next, to elucidate the dynamics of SH3BP5L, RAB11A, and KIF5B interactions, we performed kymograph tracking analysis of cells transfected with mCherry-SH3BP5L, iRFP-RAB11A, and GFP-KIF5B using confocal microscopy. This analysis revealed the enrichment of SH3BP5L followed by the recruitment of KIF5B to nascent RAB11A^+^ vesicles. These vesicles emerged from the perinucleus-positioned organelles and were directed toward the plasma membrane (Figure 3, K and L, and Supplemental Methods). Together, these data indicate that SH3BP5L activated RAB11A, which in turn promoted the recruitment of the anterograde motor protein KIF5B, suggesting a role for SH3BP5L in coordinating cargo vesicle recycling to the plasma membrane.

### SH3BP5L overexpression boosts plasma membrane–directed recycling of active integrin β1 in TNBC cells.

To assess the function of SH3BP5L in activating cargo recycling, we used a high-content imaging (HCI) pipeline. This in silico method infers 3D subcellular distribution by combining fluorescence prediction and segmentation (37, 38) (Supplemental Figure 5A). The prediction utilizes a neural network trained to recognize subcellular compartments in bright-field images, without the need for antibody labeling of each compartment. This approach circumvented the technical challenges of spectral separation in multicolor fluorescence imaging. The published training protocol was applied to mesenchymal MDA-MB-231 cells as well as to more basal-like MDA-MB-468 cells to recognize subcellular membranes associated with Golgi (RCAS1), the lysosome (LAMP1), recycling compartments (RAB11A), mitochondria (MitoTracker dye), and the plasma membrane (Cell Mask dye). After verifying the accuracy of the prediction (Supplemental Figure 5B), we used this method to compare the subcellular localization of SH3BP5L and its paralog SH3BP5 in MDA-MB-231 cells. Both proteins were enriched at the plasma membrane, consistent with their role as activators of RAB11-dependent anterograde transport. However, SH3BP5 showed stronger localization to mitochondria, whereas SH3BP5L was more enriched in the perinuclear RAB11^+^ recycling compartment (Supplemental Figure 5C). These differences point to functional specialization between the 2 paralogs.

**Figure 4:**
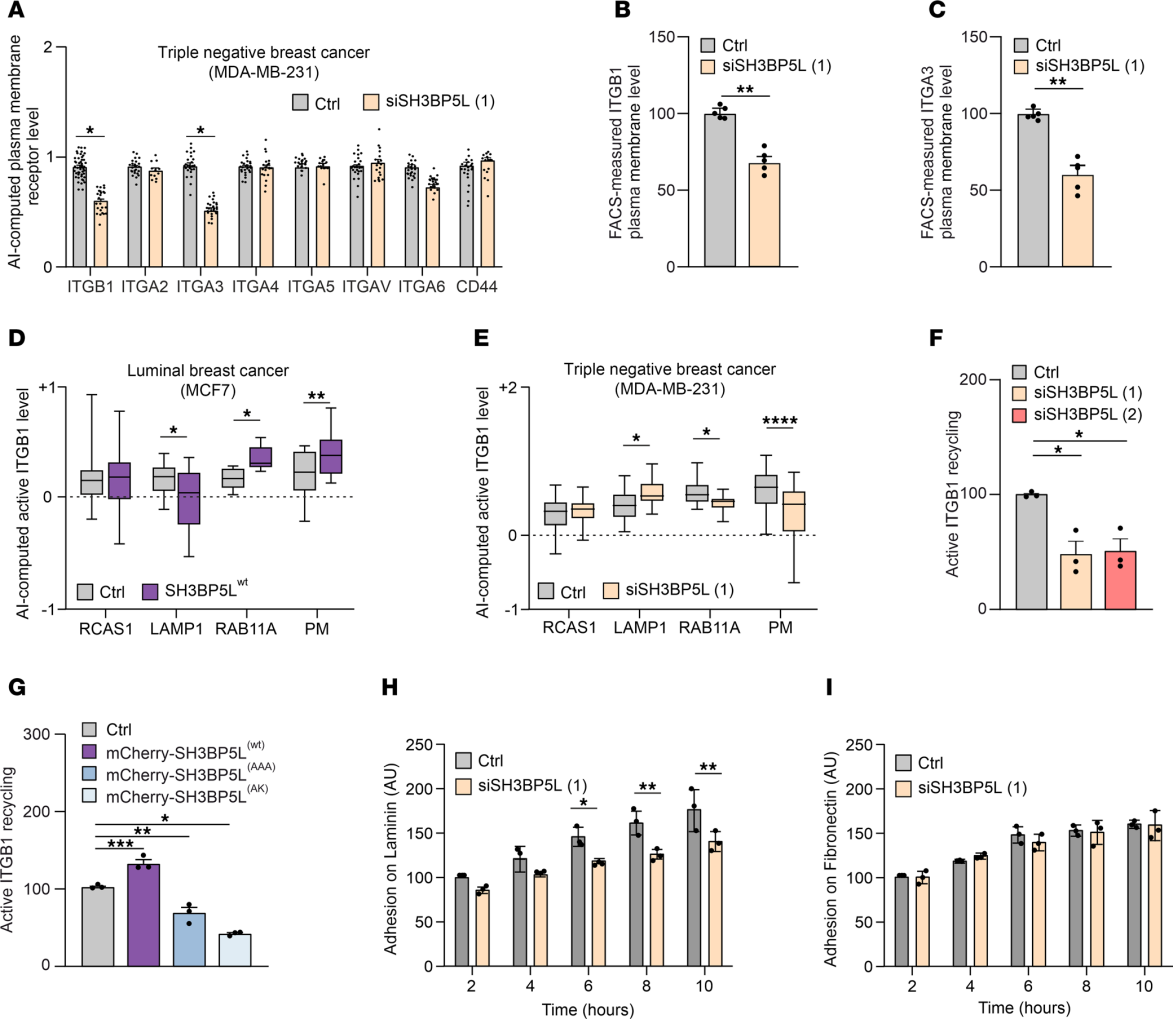
SH3BP5L boosts ITGB1 recycling. (**A**) AI-driven quantification of different integrin subunits and CD44 at the plasma membrane of MDA-MB-231 cells upon SH3BP5L depletion. *n* ≥12 cells from 3 independent experiments. (**B** and **C**) FACS quantification of plasma membrane ITGB1 (**B**) and ITGA3 (**C**) in MDA-MB-231 cells upon SH3BP5L depletion. (**D**) AI-driven quantification of active ITGB1 (with mAb 9EG7) subcellular distribution in MCF7 cells overexpressing SH3BP5L^(WT)^. *n* = 22 control cells, *n* = 18 SH3BP5L^(WT)^ cells. (**E**) AI-driven quantification of active ITGB1 (mAb 9EG7) subcellular distribution in MDA-MB-231 cells depleted of SH3BP5L. *n* = 43 control cells, *n* = 37 siSH3BP5L cells. (**F** and **G**) FACS quantification of active ITGB1 (mAb 9EG7) recycling at 30 minutes in MDA-MB-231 cells transfected with 2 different SH3BP5L siRNAs or (**F**) with mCherry, mCherry-SH3BP5L^(WT)^, mCherry-SH3BP5L^(AAA)^, or mCherry-SH3BP5L^(AK)^ constructs (**G**). (**H** and **I**) Real-time adhesion assay of MDA-MB-231 cells transfected with control or SH3BP5L siRNA and seeded on laminin (**H**) or fibronectin (**I**). Data represent the mean of at least 3 independent experiments ± SEM. *P < 0.05, **P < 0.01, ***P < 0.005, and ****P < 0.001, by 1- or 2-way ANOVA followed by Bonferroni post hoc test as appropriate.

Next, we used the HCI pipeline to functionally test if loss of SH3BP5L affects the RAB11-dependent recycling of adhesion receptors involved in metastasis. For example, BC cell motility relies on the efficient endocytosis and recycling of integrin heterodimers, which form a recycling loop between the endosomal compartment and the plasma membrane. The endosomal compartment processes endocytosed cargos from the cell surface, directing them either for degradation or recycling to the plasma membrane (39, 40). Similarly, other key adhesion receptors like CD44 control metastasis (41) and require RAB11 to recycle back once endocytosed (42). Therefore, we focused our attention on CD44 and integrin subunits such as β1, α2, α3, α4, α5, αV, and α6. SH3BP5L KD led to a selective reduction of integrin β1 (ITGB1) and integrin α3 (ITGA3) at the plasma membrane, whereas the localization of other integrins and CD44 remained unaffected (Figure 4A and Supplemental Methods). To further validate the accuracy of this approach, the decrease in plasma membrane content of ITGB1 and ITGA3 in cells where SH3BP5L was downregulated was confirmed by FACS staining (Figure 4, B and C). These findings demonstrate that SH3BP5L selectively regulated β1 and α3 integrin recycling and confirm the ability of the HCI pipeline to faithfully capture the intracellular distribution of ITGB1 in BC cells.

We next examined whether SH3BP5L regulates recycling of the active conformation of β1 integrin, as only the ligand-binding active pool engages the extracellular matrix and transduces signals required for migration (43). To validate the ability of the HCI pipeline to detect active ITGB1, fixed MDA-MB-231 cells were labeled with antibodies recognizing active (9EG7), inactive (mAb13), or total ITGB1 (P5D2) (44). Consistent with previous reports (43, 45), total ITGB1 in control cells was primarily localized to the plasma membrane, while active ITGB1 was also present in intracellular compartments (Supplemental Figure 5D). The unrelated cytoplasmic protein mCherry showed no specific compartmentalization (Supplemental Figure 5D). With this validation of the HCI pipeline, we further investigated whether induced overexpression of SH3BP5L alters active ITGB1 distribution in MCF7 cells, a nonmetastatic luminal BC cell line that expresses physiological levels of this GEF (Figure 1F). In these cells, SH3BP5L overexpression increased active ITGB1 in the RAB11A^+^ compartment and at the plasma membrane, while reducing its accumulation in lysosomes as indicated by fewer LAMP1^+^ vesicles (Figure 4D and Supplemental Methods).

Conversely, SH3BP5L KD in the TNBC cell lines MDA-MB-231 and MDA-MB-468 reduced active ITGB1 levels in the RAB11A^+^ compartment and at the plasma membrane, while promoting its accumulation in LAMP1^+^ lysosomes (Figure 4E and Supplemental Figure 5E). These results indicate that SH3BP5L promotes plasma membrane delivery of active ITGB1 by controlling its intracellular trafficking. To directly assess recycling dynamics, we performed antibody-based assays using 9EG7 and measured surface reexposure of active ITGB1 by flow cytometry (43). SH3BP5L depletion markedly impaired active ITGB1 recycling in 2 TNBC cell lines (Figure 4F, Supplemental Figure 5, F and G, and Supplemental Figure 6A). We observed a similar defect when the GEF activity of SH3BP5L was abolished using catalytically inactive mutants (Figure 4G and Supplemental Figure 6B).

Finally, to evaluate the functional consequences of this defect, we examined cell adhesion to specific ECM ligands. SH3BP5LKD in MDA-MB-231 cells significantly reduced adhesion to laminin, a known ligand for α3β1 activation, but had no effect on fibronectin, which primarily engages other β1-containing integrin heterodimers (Figure 4, H and I). Together, these results demonstrate that SH3BP5L controlled the recycling of α3β1 integrin in a GEF-dependent manner, providing a mechanistic link to its role in sustaining TNBC cell adhesion and metastatic potential.

### SH3BP5L couples RAB11A activation to TNBC invasion in fish, mouse, and patient-derived models.

**Figure 5:**
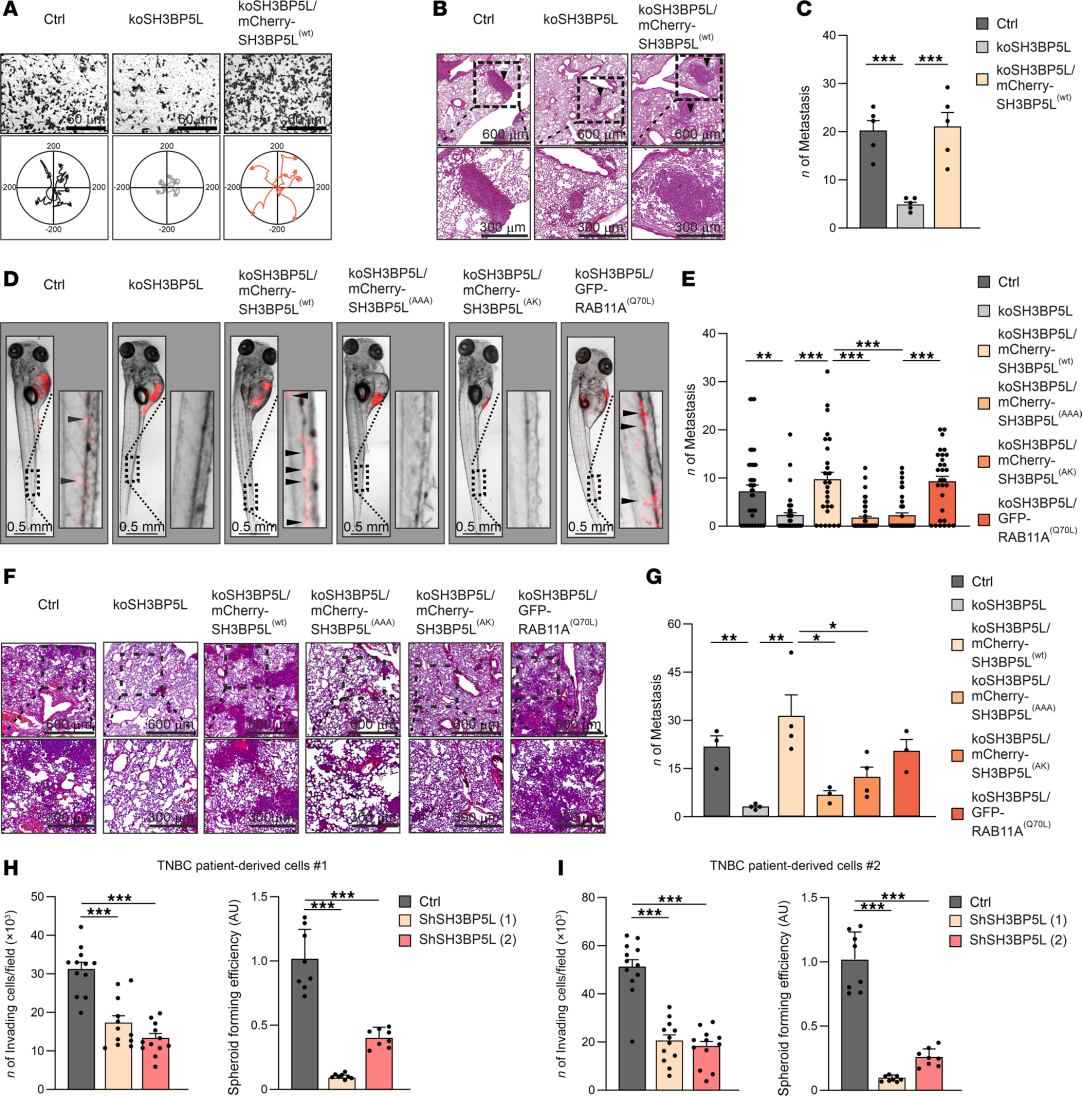
SH3BP5L enhances RAB11A-mediated invasion and metastasis. (**A**) Representative image of Transwell (upper) and single-cell tracking (bottom) assay of MDA-MB-231 cells KO for SH3BP5L cells and transfected with mCherry-SH3BP5L^(WT)^ (koSH3BP5L/mCherry-SH3BP5L^(WT)^). For cell tracking, *n* = 25 (Ctrl), *n* = 36 (koSH3BP5L), *n* = 21 (koSH3BP5L/mCherry-SH3BP5L^(WT)^) cells, respectively. Scale bars: 600 μm and 300 μm (enlarged insets). (**B** and **C**) Representative images (**B**) and relative quantification (**C**) of H&E staining of lung metastases in NSG mice injected in the tail vein with koSH3BP5L in MDA-MB-231 cells and transfected with mCherry-SH3BP5L^(WT)^ (koSH3BP5L/mCherry-SH3BP5L^(WT)^). Black arrowheads indicate metastatic foci. Each dot in the graph in **C** is representative of an injected mouse (*n* = 5). Scale bars: 60 μm. (**D** and **E**) Representative images (**D**) and relative quantification (**E**) of metastasized foci in zebrafish injected with MDA-MB-231 cells with koSH3BP5L and transfected with mCherry-SH3BP5L^(WT)^ (koSH3BP5L/SH3BP5L^(WT)^), mCherry-SH3BP5L^(AAA)^ (koSH3BP5L/SH3BP5L^(AAA)^), mCherry-SH3BP5L^(AK)^ (koSH3BP5L/SH3BP5L^AK^), or GFP-RAB11A^(Q70L)^ (koSH3BP5L/RAB11A^Q70L^). Arrowheads indicate metastatic foci in zebrafish tails. Each dot in the graph in **E** is representative of an injected zebrafish (*n* ≥30). Scale bars: 0.5 mm. (**F** and **G**) Representative images (**F**) and relative quantification (**G**) of H&E staining of lung metastases in NSG mice orthotopically injected with MDA-MB-231 cells with koSH3BP5L and transfected with mCherry-SH3BP5L^(WT)^ (koSH3BP5L/SH3BP5L^(WT)^), mCherry-SH3BP5L^(AAA)^ (koSH3BP5L/SH3BP5L^(AAA)^), mCherry-SH3BP5L^(AK)^ (koSH3BP5L/SH3BP5L^(AK)^), or GFP-RAB11A(Q70L) (koSH3BP5L/RAB11A^(Q70L)^). Each dot in the graph in **G** is representative of an injected mouse (*n* ≥3). Scale bars: 600 μm and 300 μm (enlarged insets). (**H** and **I**) In vitro Matrigel Transwell invasion assay (left) and spheroid-forming assay (right) of cells derived from 2 patients with TNBC (patient 1, **H**; patient 2, **I**) transduced with either control or 2 independent lentiviral shRNAs targeting SH3BP5L (left; *n* = 12 fields per group from 3 independent experiments; right: *n* = 8). Data represent the mean of at least 3 independent experiments ± SEM. *P < 0.05, **P < 0.01, and ***P < 0.005, by 1-way ANOVA.

We next sought to investigate the role of SH3BP5L in TNBC metastasis in vivo. First, we generated a SH3BP5L-KO (koSH3BP5L) MDA-MB-231 cell line using CRISPR/Cas9, along with a control cell line, in which SH3BP5L expression was rescued by expressing WT SH3BP5L (Supplemental Figure 7A). As previously observed (Supplemental Figure 3H), reduction of SH3BP5L expression did not affect cell proliferation but significantly decreased cell migration in vitro (Figure 5A, Supplemental Figure 7, B–D, and Supplemental Methods). We then injected these engineered KO cells into the tail vein of NSG mice. Five weeks after injection, koSH3BP5L cells exhibited a marked reduction in lung metastasis compared with WT cells or koSH3BP5L cells rescued by mCherry-SH3BP5L^(WT)^ expression (Figure 5, B and C). We repeated the experiment in a zebrafish embryo model (46–48), which allowed us to perform live imaging of cancer cell dissemination. In agreement with our observations in mice, koSH3BP5L MDA-MB-231 and MDA-MB-468 cells exhibited a significantly reduced incidence of metastasis and fewer disseminated foci in zebrafish tails compared with WT cells. Reintroduction of SH3BP5L expression in koSH3BP5L cells restored metastatic spreading to levels comparable to those of wild-type controls (Figure 5, D and E, and Supplemental Figure 7, E and F).

Next, we performed rescue experiments using WT and mutant forms of SH3BP5L, as well as WT and constitutively active RAB11A, to determine whether SH3BP5L GEF activity influences in vivo invasion. We injected koSH3BP5L MDA-MB-231 cells into the yolk sac of zebrafish larvae and quantified their spread to the tail region. Consistent with the Transwell assay results (Supplemental Figure 3, F and G), we observed reduced cell spreading in larvae injected with koSH3BP5L cells and rescue of this defect upon introduction of an active RAB11A^(Q70L)^ mutant into the KO cells (Figure 5, D and E). In contrast, reintroducing WT, but not GEF-deficient, SH3BP5L, into koSH3BP5L cells restored distant spreading. These experiments demonstrate that cell dissemination was dependent on SH3BP5L GEF activity toward RAB11A (Figure 5, D and E). We observed similar results in NSG mice as, 5 weeks after injection, restoring RAB11A activity increased the number of lung metastases in SH3BP5L-KO cells, whereas impairing SH3BP5L GEF function reduced lung metastases (Figure 5, F and G).

Finally, to assess the clinical relevance of SH3BP5L expression, we used 2 TNBC patient-derived xenograft (PDX) models along with a patient-derived tumor spheroid (PDTS) assay, which is commonly used as a functional readout for tumor-initiating cell (TIC) activity in BC (49). SH3BP5L KD markedly impaired cell invasion and spheroid formation but had no effect on proliferation (Figure 5, H and I, and Supplemental Figure 7, G and H). As TIC-like activity is frequently linked to metastatic dissemination, these findings align with our in vitro and in vivo models showing that the GEF activity of SH3BP5L promoted TNBC migration, invasion, and distant colonization.

## Discussion

The metastatic progression of BC remains a major clinical challenge, particularly in subtypes with limited therapeutic options. In this study, we identified SH3BP5L as a GEF for RAB11A that is upregulated in BCs with aggressive features. Analysis of large patient cohorts demonstrated that high SH3BP5L expression correlated with advanced tumor stage, high grade, and poor outcomes. Importantly, SH3BP5L^hi^ tumors were enriched not only in TNBC but also in HER2^+^ cancers, and in both subtypes high SH3BP5L expression was associated with reduced DMFS. These findings establish SH3BP5L as a clinically relevant marker of poor prognosis across aggressive BC subtypes. Nonetheless, our mechanistic studies focused on TNBC for 3 reasons. First, transcriptomics analyses in both METABRIC and TCGA consistently demonstrated increased SH3BP5L expression in TNBC compared with luminal tumors, and high expression was specifically linked to poor RFS in TNBC. Second, TNBC is the BC subtype most prone to early and frequent metastatic dissemination (1). Third, unlike HER2^+^ cancers, where multiple targeted therapies have markedly improved patient outcomes, TNBC remains without comparably effective options.

RAB11 activity assays confirmed that SH3BP5L activated RAB11A in TNBC cells, while coimmunoprecipitation assays demonstrated a specific interaction between active RAB11A and KIF5B, a kinesin motor protein essential for anterograde intracellular cargo transport. In vitro binding assays further showed that KIF5B exhibited a higher affinity for GTP-bound, rather than GDP-bound, RAB11A, supporting a model in which SH3BP5L activates RAB11A, enabling its GTP-bound form to preferentially associate with KIF5B. KIF5B had previously been identified as a RAB11 interactor through BioID analysis (24), but our data now provide direct functional evidence that SH3BP5L-mediated RAB11 activation facilitates this interaction. AlphaFold3 prediction and imaging data aligned with this model, demonstrating that SH3BP5L-mediated activation of RAB11A enabled its association with KIF5B and promoted vesicle trafficking from the perinuclear region to the plasma membrane. Consistent with this, our proteomics analysis did not detect interactors typically associated with the inactive GDP-bound state of RAB11A. In particular, KIF5A, a kinesin known to bind RAB11A-GDP (50), was absent, indicating that SH3BP5L reshaped the RAB11A interaction landscape by maintaining it in the active GTP-bound state.

Dynamic kymograph tracking further reinforced this model by revealing the temporal organization of the SH3BP5L-RAB11A-KIF5B complex in living cells. SH3BP5L promoted the formation of anterograde vesicles containing integrins, including ITGB1, and SH3BP5L KD reduced steady-state levels of active ITGB1 at the plasma membrane. This selective effect on active ITGB1 recycling was accompanied by impaired migration and spreading of SH3BP5L-deficient TNBC cells. Since ITGB1 recycling is known to facilitate cancer cell motility and invasion (51), our findings position SH3BP5L as a central regulator that couples RAB11 activation to integrin recycling through KIF5B in TNBC cells.

Mechanistically, these results converge with our integrin trafficking studies showing that SH3BP5L selectively controlled the recycling of the laminin-binding α3β1 heterodimer. Although the recycling of other integrins appeared unaffected, this specificity was sufficient to drive major changes in cell migration and metastatic dissemination. Indeed, α3β1 has been repeatedly implicated as a critical driver of invasion and poor prognosis in basal-like and TNBCs (52, 53). Its engagement with laminin, rather than with other ECM components, provides essential cues for directional motility and metastatic progression. In support of this, SH3BP5L-depleted cells exhibited impaired adhesion and spreading on laminin, but not on fibronectin, indicating that loss of α3β1 recycling selectively disabled the migratory machinery relevant to metastasis. These findings are consistent with prior reports documenting that functional blockade or genetic silencing of integrin α3 is sufficient to suppress adhesion, migration, and metastatic invasion in BC models (54–58). Together, our results and previous work highlight α3β1 as a pivotal effector whose trafficking is controlled by SH3BP5L/RAB11A signaling. Future studies will be needed to define the molecular basis of this specificity and clarify how SH3BP5L discriminates α3β1 from other β1-containing integrins during recycling.

The functional significance of this pathway was first demonstrated in vivo, where zebrafish and mouse metastasis models showed that SH3BP5L GEF activity is required for dissemination and colonization. This conclusion was further reinforced by ex vivo validation in short-term cultures derived from tumors of patients with TNBC, which preserved the heterogeneity of the parental lesions and provided a clinically relevant platform to test SH3BP5L dependency. In these patient-derived systems, SH3BP5L KD markedly impaired invasion and spheroid formation while leaving proliferation unaffected. The ability to form spheroids in nonadherent conditions is commonly used as a functional readout of tumor-initiating potential (49, 59), a property frequently associated with metastatic dissemination (60). Accordingly, the reduced spheroid formation observed after SH3BP5L silencing was consistent with the impaired invasion and colonization seen in vivo. Taken together with the retrospective cohort analysis, these findings establish SH3BP5L as both a prognostic marker and a functional driver of TNBC aggressiveness.

From a therapeutic perspective, our findings highlight SH3BP5L as an attractive target to counteract BC dissemination. Unlike direct integrin antagonists (40), which have largely failed in clinical trials due to redundancy and compensatory mechanisms at the receptor level (61), inhibition of SH3BP5L would act upstream by blocking integrin recycling, thereby indirectly reducing tumor cell adhesion and dissemination. One potential concern for this strategy is the presence of the close paralog SH3BP5. Yet our data indicate that the 2 proteins fulfilled clearly distinct functions: SH3BP5L was enriched in the perinuclear RAB11^+^ recycling compartment, bound KIF5B, and sustained integrin recycling and invasion, whereas SH3BP5 predominantly localized to mitochondria and lacked these activities. High SH3BP5L expression correlated with reduced survival in BC cohorts, while SH3BP5 showed the opposite trend. These spatial differences, together with the higher catalytic efficiency of SH3BP5L toward RAB11A, offer a plausible mechanistic explanation for the opposing clinical associations of the 2 paralogs. This divergence makes it unlikely that SH3BP5 could compensate for SH3BP5L inhibition, but at the same time it emphasizes the importance of achieving stringent isoform selectivity. Encouragingly, selective pharmacological targeting of GEFs is feasible, and there is increasing recognition of their druggability in different disease contexts (62). A prominent precedent is provided by brefeldin A, which selectively inhibits only a subset of the ARF1 GEF family by stabilizing an abortive ARF-GEF intermediate, while other ARF1 GEFs remain unaffected (63). Although no inhibitors of SH3BP5L currently exist, our genetic proof of concept provides a compelling rationale for developing small molecules or peptidomimetics capable of selectively blocking SH3BP5L activity or its interaction with RAB11A.

**Figure 6:**
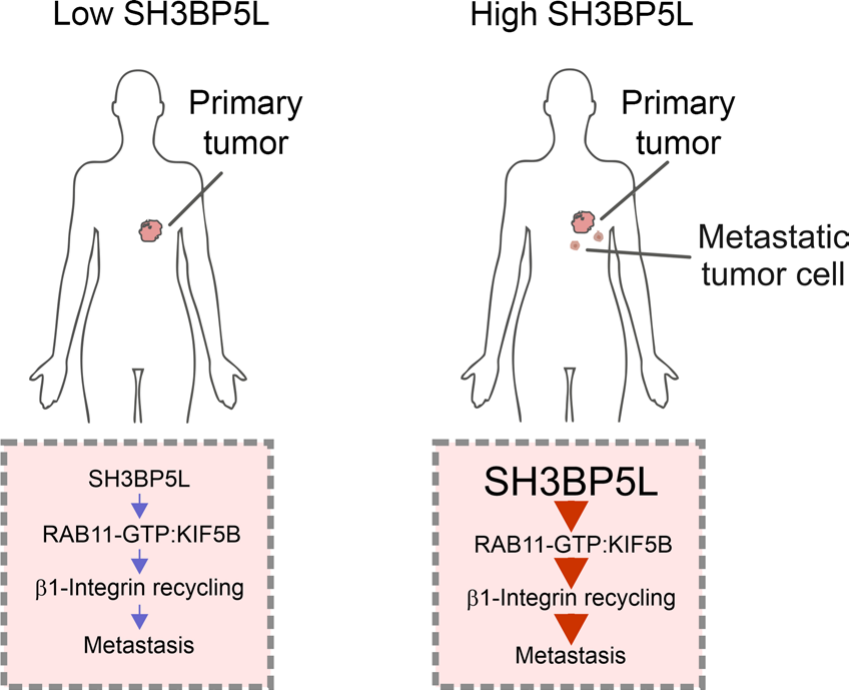
Graphical abstract. Schematic representation illustrates that in models expressing high levels of SH3BP5L, SHBP5L interacts with activated RAB11A (RAB11-GTP), which binds KIF5B to facilitate active ITGB1 recycling, ultimately driving metastatic dissemination.

In conclusion, we identify SH3BP5L as what we believe to be the first RAB11 GEF with a defined role in BC metastasis. High SH3BP5L expression marked aggressive tumors and correlated with poor outcomes in HER2^+^ and TNBC. Mechanistically, SH3BP5L coupled RAB11A activation to KIF5B-mediated transport of α3β1 integrins, driving invasion and dissemination. Functional validation in cell lines, animal models, and patient-derived cultures revealed SH3BP5L as both a prognostic determinant and a therapeutic vulnerability in TNBC. Targeting SH3BP5L-dependent trafficking may thus offer a new strategy to counteract metastatic progression in a subtype of BC that urgently requires better treatment options (Figure 6).

## Methods

### Sex as a biological variable.

Male BC comprise less than 1% of all BC cases (64). This study, therefore, included only female patients and mice.

### Patients.

A cohort of 1,590 patients with operable BC was evaluated using IHC on a tissue microarray. These patients underwent surgery at the European Institute of Oncology (IEO) in Milan between 1997 and 2000. As previously reported (29), antibody specificity was validated using SH3BP5L-KO cells, and staining intensity was assessed in a blinded manner by a pathologist. IHC staining was scored on a scale from 0 to 3. Samples with a staining intensity of 2 or greater in either the plasma membrane or the cytoplasm were classified as SH3BP5L^hi^.

### Public BC cohort analysis.

Gene expression data for TCGA–Breast Cancer (TCGA-BRCA) (65) were retrieved from cBioPortal. Of 1,082 tumors with transcriptomics data, we excluded 2 samples with nonadenocarcinoma histology. Molecular subtypes were inferred from transcriptomes using the SCMOD2.robust algorithm (66). METABRIC gene expression and clinicopathological data (26) were retrieved from cBioPortal. Of the 1,980 tumors, we excluded 6 samples with nonadenocarcinoma histology. Subtypes were assigned on the basis of estrogen receptor and human epidermal growth factor receptor 2–IHC (HER2-IHC) and HER2-SNP6 array data; missing annotations were inferred with SCMOD2-robust. Microarray-based gene expression and follow-up data harmonized across multiple datasets (67) were obtained from KM-plotter. SH3BP5L and SH3BP5 expression data were available for 2,032 and 4,929 patients, respectively.

For survival analyses, METABRIC and KM-plotter datasets were merged into a Composite Breast Cancer cohort. Within each source, log_2_-transformed probe intensities were standardized to *z* scores. Subgroup analyses were performed according to PAM50 subtype. Associations between SH3BP5 or SH3BP5L expression and RFS were evaluated using Cox proportional hazards models. Gene expression was modeled as a continuous variable (linear or penalized-spline terms) and as a binary variable (upper tertile “high,” lower 2 tertiles “low”). Significance was assessed with 2-sided log-rank tests.

### Cell lines.

Cell lines were bought from the American Type Culture Collection (ATCC). The MCF7 cell line was cultured in Gibco’s RPMI-1640 medium, supplemented with 10% FBS and 1% penicillin-streptomycin (10,000 U/mL). The SKBR3, MDAMB468, T47D, BT474, BT549, MDA-MB-231, HEK293T, HCC1937, HCC38, HCC70, HCC1143, and HCC1806 cell lines were cultured in DMEM and supplemented with 10% FBS and 1% penicillin-streptomycin (10,000 U/mL). Two independent patient-derived TNBC cell populations (PDX models), previously stratified for high SH3BP5L expression by IHC analysis, were cultured in a 1:1 mixture of DMEM and Ham’s F12 medium, supplemented with 2 mM l-glutamine, 5 μg/mL insulin, 0.5 μg/mL hydrocortisone, 2% B27, 20 ng/mL EGF and FGF and 4 μg/mL heparin. All the cell lines were cultured in a humidified incubator (Thermo Fisher Scientific) with 5% CO_2_ at 37°C. All cell lines described had been tested for mycoplasma contamination.

### Proliferation assay.

Cells were seeded in a sterile 96-well plate (5,000 cells/well) in 100 μL complete culture medium and incubated at 37°C and 5% CO_2_. Cells were transfected with siRNAs, and cell proliferation was monitored for 5 days following the standard Incucyte protocol for proliferation.

For the growth assays performed on the 2 independent patient-derived TNBC cell populations, cells were plated at a density of 50,000 cells per well in 24-well plates. Cell growth was assessed by cell counting. Time 0 was considered the time of the initial seeding.

### Colony spheroid formation assay.

The colony spheroid formation assay in methylcellulose was performed on the 2 independent patient-derived TNBC cell populations, as previously described (49). Briefly, single-cell suspensions (1,000 cells/mL) were plated in 24-well plates under nonadherent conditions in stem cell medium (1:1 mixture of DMEM and Ham’s F12 medium, supplemented with 2 mM l-glutamine, 5 μg/mL insulin, 0.5 μg/mL hydrocortisone, 2% B27, 20 ng/mL EGF and FGF, and 4 μg/mL heparin) containing 1% methylcellulose (MilliporeSigma) and incubated (humidified 5% CO_2_, 37°C). Colonies were manually counted after 12 days. The spheroid-forming efficiency (SFE) percentage (number of spheres/number of plated cells × 100) was then calculated.

### Transwell and single-cell tracking assays.

To perform the cell migration assay, 3 × 10^4^ cells were suspended in serum-free medium and seeded into the upper chamber of a 24-well Boyden chamber (8 μm; Corning). Medium (500 μL) with 10% FBS was added in the bottom chamber. The nonmigrated/invaded cells were removed after 48 hours by cotton swabs, and cells that migrated/invaded through the membranes were fixed with 4% PFA for 20 minutes and stained with 0.5% crystal violet for 10 minutes and eventually washed 3 times with distilled water (MilliporeSigma). Images of 5 random fields for each membrane were captured using a light microscope (Leica, Olympus, BX41). Migrated/invaded cells were counted using ImageJ software (NIH). For the single-cell tracking assay, transfected MDA-MB-231 cells were seeded on a μ-Slide 8 Well (Ibidi) and cultured overnight. Cells were maintained at 37°C and 5% CO_2_, and cell migration was monitored using a Leica TCS-II SP5 confocal microscope (×10 objective). Cells were imaged every 10 minutes over a 16-hour period. To assess cell migration, speed, and distance, single cells were manually tracked using the Manual Tracking plug-in from ImageJ.

For the 2 independent patient-derived TNBC cell populations, a Transwell invasion assay was performed using PET membrane inserts (BRAND 24-well Cell Culture Insert, PET membrane, no. BR782711, Merck) covered with 20 μL Matrigel/PBS (1:2) as previously described (68). Briefly, 1 × 10^5^ cells were seeded in growth factor–free medium in the upper chamber of the Transwell insert. Complete medium supplemented with HGF (25 ng/mL) was added to the lower part of the Transwell. After approximately 36 hours of incubation, the Transwell inserts were removed and the cells were fixed in the Transwell insert with 4% paraformaldehyde (PFA) for 15 minutes. Cells were then washed with water to remove the formaldehyde. Then, using a sterile cotton swab, cells that had not migrated through the membrane were scraped off the top of the Transwell insert and stained with DAPI plus 0.1% Triton X-100 in PBS for 30 minutes. Images corresponding to the entire area of the Transwell were collected with a Nikon Ti-2 microscope using a ×10 objective, and the number of invaded cells was analyzed using Fiji software.

### Cell-spreading assay.

Real-time adhesion of MDA-MB-231 control cells or SH3BP5L-silenced (siSH3BP5L cells was monitored using a real-time electrical impedance–based system (xCELLigence, Agilent Technologies). In brief, the bottom side of the E-Plate 16 was coated with 0.5 mg/mL of either laminin (Mouse Laminin, Merck/MilliporeSigma, L2020) or fibronectin (Human Fibronectin, R&D Systems, 1918-FN) for 1 hour at room temperature. Then, the protein-coated plate was washed with PBS and incubated with 3% BSA solution in PBS for 1 hour at 37°C. Cells were detached by means of trypsin-EDTA and resuspended to a final concentration of 8,000 cells/100 mL. The BLANK step was started to measure the background impedance of the cell culture medium, which was then used as a reference impedance to calculate CI values. Cell suspension (100 mL; *n* = 8,000 cells) was then added to each well. The E-Plate 16 was placed in the RTCA DP (Agilent Technologies) instrument equilibrated in a CO_2_ incubator. Cell adhesion was continuously monitored using the RTCA DP instrument. The mean, SD, and *P* value were calculated for the CI data and exported from the RTCA instrument, including the technical replicates of each experimental condition at each time point.

### In situ PLA.

A PLA was performed using the Duolink Proximity Ligation Assay kit (Duolink, Olink BioScience). PFA-fixed cells were incubated for 1 hour with a pair of primary antibodies, each produced in different species, against the putative interacting partners. Duolink minus and plus probes were used to detect antibody-labeled proteins. Samples were examined with a Leica TSC-II SP8 confocal microscope.

### Protein and pull-down analyses.

For direct protein detection, cells were lysed in buffer (120 mM NaCl, 50 mM Tris-HCl pH 8.0, 1% Triton X-100, protease inhibitors, 50 mM NaF, 1 mM Na_3_VO_4_) and cleared by centrifugation (16,200*g*, 10 minutes, 4°C). Protein concentration was measured by Bradford assay. For immunoprecipitation, cells were lysed in 50 mM Tris-HCl, pH 8.0, 150 mM NaCl, 1% NP-40, 1 mM EDTA, 10% glycerol, and protease/phosphatase inhibitors. Lysate (1 mg) was incubated with either 1 μg antibody for 1.5 hours at 4°C, followed by 15 μL protein G-sepharose for 30 minutes, or with 15 μL GFP-Trap Magnetic Agarose (ChromoTek) for 2 hours. Complexes were pelleted (3,000*g* for 1 minute), washed 6 times, and eluted in 30 μL Laemmli buffer. For active Rab11 pull-downs, lysates prepared as above, plus 10 mM MgCl_2_, were incubated with 25 μL GST-FIP3 (specific for active Rab11A) (69) bound to glutathione agarose (GE) for 1 hour at 4°C, washed 4 times, and eluted in Laemmli buffer.

For RAB11 effector pull-downs, GST-RAB11A was produced in *E. coli*, induced with 0.1 mM IPTG, purified on glutathione resin, dialyzed, and stored at –80°C. Fifty micrograms of GST-RAB11A or GST was coupled to glutathione agarose (1 hour, 4°C), stripped of nucleotides with buffer A (four 20-minute cycles at 25°C), and loaded with 2 mM GDP or GTPγS in buffer B (1 hour at 25°C). After washing in buffer C, beads were incubated with purified KIF5B-HA for 1 hour in GST-binding buffer, washed, and eluted by boiling in Laemmli sample buffer. Supernatants and pull-down eluates were analyzed by immunoblotting with the indicated primary and HRP-conjugated secondary antibodies and detected by ECL (BD). GAPDH or vinculin served as loading controls. Uncropped blots are provided in Supplemental Figure 8.

### Immunofluorescence.

Immunofluorescence was performed on 4% PFA-fixed cells, followed by permeabilization with 0.1% Triton X-100 for 5 minutes and blocking in 1% BSA for 20 minutes. Next, permeabilized cells were incubated with the indicated primary antibodies and fluorescent secondary antibodies for 60 minutes each. Cells were examined with a Leica TSC-II SP8 confocal microscope. Raw images were digitally processed to normalize the background and enhance the contrast.

### Proteomics analysis of SH3BP5L-binding partners.

HEK293 cells (0.8 × 10^6^ cells) were transfected with GFP (mock) or GFP- SH3BP5L (full-length), 16-hour post-transfection cells were starved without serum for another 2 hours as the serum-free group, and cells after the re-addition of DMEM with 10% FBS for 40 minutes were designated as the serum-fed group. Upon serum stimulation, internalized integrins were recycled back to the plasma membrane to support cell migration. This behavior is consistent with previous work from our and other groups (32–35) showing that serum provides multiple cues — including growth factors and ECM components such as fibronectin — that trigger endocytosis and recycling. Next, cells were washed in PBS buffer and lysed in 200 μL lysis buffer (10 mM Tris/Cl pH 7.5, 150 mM NaCl, 0.5 mM EDTA, 0.5% Nonidet P40 substitute). Total protein (1.5 mg) was used for affinity purification using 10 μL GFP-Trap MA beads (ChromoTek). Samples were gently shaken using tube rotators and incubated for 1 hour at 4°C. The beads were washed 3 times with lysis buffer. Bound proteins were eluted with ×2 SDS-PAGE loading buffer and sent to the Proteomics Core Facility (EMBL Heidelberg) for proteomics analysis of SH3BP5L-associated proteins.

### AlphaFold3 modeling of the KIF5B-RAB11A interaction.

AlphaFold3 searches were carried out using the AlphaFold3 server (https://alphafoldserver.com/) (36). Searches were done using a dimer of full-length KIF5B, full-length RAB11A, 2 ATP molecules, 3 magnesium ions, and either GDP or GTP. The predicted alignment error (pae) for these predictions is shown in Supplemental Figure 3H. The chain pair interface–predicted template modeling (iptm) scores were 0.47 and 0.47 for RAB11A (GTP) for binding to each of the 2 KIF5B subunits. When the AlphaFold3 search was carried out using RAB11A (GDP) the chain pair iptm scores were 0.25 and 0.26 for RAB11A (GDP) binding to each of the 2 KIF5B subunits.

### AS-RAB11A FRET analysis.

Cells were transfected with the RAB11A FRET biosensor (AS-RAB11A) and fixed in 4% PFA. Cells were washed with PBS, blocked in 1% BSA solution, and imaged using a Leica TSC-II SP8 confocal microscope ×63 objective. The FRET to cyan fluorescent protein (CFP) ratio (FRET/CFP) was quantified using ImageJ software, according to our previous work (30). For the fluorometry assay, 2 × 10^5^ HEK293T cells were plated in a 6-well plate and transfected using Lipofectamine 2000 (Invitrogen, Thermo Fisher Scientific). Thirty-six hours after transfection, cells were lysed in lysis buffer (50 mM Tris–HCl, pH 7.4, 10 mM MgCl2, 100 mM NaCl, 1% Triton X-100, proteinase inhibitors), and clarified lysates were analyzed using a HORIBA Fluoromax-4 fluorometer. The lysates were excited at 433 nm, and emission was registered between 450 and 550 nm. Direct excitation/emission of yellow fluorescent protein (Venus) at 505/525 nm was used to normalize the biosensor concentration.

### Single-cell subcellular distribution.

Large confocal fields (sized in pixels *x* = 1,848, *y* = 1,248, *z* = 64; in micrometers: *x* = 160.54, *y* = 108.42, *z* = 18.27) of MDA-MB-231, MDA-MB-468, and MCF7 cells were acquired to train label-free machine-learning models of multiple subcellular compartments (37). To train the models, cells were grown in 8-well Ibidi plates and treated with fluorescence trackers or fixed and stained with antibodies targeting RCAS1, LAMP1, and RAB11A proteins and dyes such as DAPI, Cell Mask, and MitoTracker. By comparing the model’s overall performance on the training dataset (the average correlation of the predicted images with the training ground truth, or cMax) with the testing dataset (the correlation of the predicted images with the testing ground truth), the training efficiency was evaluated (37). Next, the protein of interest (POI) was stained in a separate experiment. The label-free models, previously described (37), were inferred, segmented, and superimposed to detect single-cell borders and compute single-cell subcellular enrichment of the POI in Python.

### Active ITGB1 recycling assay.

An active ITGB1 recycling assay was performed as previously described (6, 43). In short, surface protein was labeled by anti–active ITGB1 antibody (9EG7) in HBSS for 30 minutes on ice. Unbound antibody was washed away, and cells were cultured in prewarmed medium for 1 hour at 37°C (internalization). Antibody was stripped by a brief acid wash (0.5% acetic acid, 0.5 M NaCl, pH 3.0), as recycling time 0. After 30 minutes at 37°C (inducing recycling), cells were fixed, transferred to ice, stained with Alexa Fluor 488 to detect surface active ITGB1, and subjected to flow cytometry detection. Flow cytometry was performed on a BD FACSVerse flow cytometer, and analysis was performed using FCSalyzer, version 0.9.22-alpha.

### Zebrafish strains and metastasis assay.

The WT fish strain Tuebingen was used. Adult fish were routinely maintained under a 14-hour light/10-hour dark photo period at approximately 28°C and were bred and genotyped according to standard procedures. Eggs were generated by natural mating and, following fertilization, were collected, treated, and maintained under a 12-hour light/12-hour dark photo period at 28°C. Embryos were treated with 0.003% 1-phenyl-2-thiourea (PTU) (no. P7629, MilliporeSigma) at 24 hours post fertilization (hpf) to prevent the formation of melanin pigment, which could interfere with the visualization of fluorescence in the metastatic assay. Embryos and adult fish were sacrificed with a tricaine overdose. For zebrafish xenotransplantation, At 48 hpf, WT zebrafish embryos were anesthetized with 0.04 mg/mL tricaine (MilliporeSigma) before cancer cell injection. Approximately 300 Vybrant DiI–labeled (red) tumor cells were injected into the yolk sac of each embryo, and zebrafish were maintained in E3 medium for 1 hour at 28°C. After confirmation of a visible cell mass at the injection site, zebrafish were maintained at 30°C for 72 hours in standard embryo medium supplemented with 0.003% PTU, 1 g/L glucose, and 5 mmol/L l-glutamine. Images were acquired with a Zeiss Observer-Z1 microscope (×10 objective). Because of the large size of the embryos, in the representative images, sequential images of each embryo were acquired and composed to show the whole embryo.

### Murine metastatic model.

Female BALB/c NSG mice (8–12 weeks of age) weighing between 18 and 20 g were housed at 22°C ± 5°C in a 12-hour light/12-hour dark cycle and fed rodent chow and water freely, MDA-MB-231 cells (10^5^ cells) were injected into the tail vein. Mice were sacrificed after 5 weeks, and lungs were collected for H&E staining to count lung metastases.

### Statistics.

GraphPad Prism software or R software (version 4.1.2) was used for statistical analysis. Significance was calculated with Student’s *t* test, 1- or 2-way ANOVA followed by Bonferroni’s post hoc analysis, or Mantel-Cox log-rank test where appropriate. Differences in proportions were tested with Fisher’s exact test in all 2 × 2 tables and with Pearson’s χ^2^ test in larger contingency tables. Values are reported as the mean ± SEM. A *P* value of less than 0.05 was considered statistically significant (*), *P* of less than 0.01 highly significant (**), and *P* of less than 0.001 extremely significant (***).

### Study approval.

The study on the human BC cohort was approved by the IRB of the European Institute of Oncology (Milan, Italy), and informed consent was obtained from all participants. All procedures using zebrafish (Danio Rerio) were authorized by the Ethics Committee of the University of Torino and the Italian Ministry of Health (authorization no. 707/2022-PR issued on November 15, 2022 and authorization no. 22/2017-UT, issued on November 20, 2017). Studies involving all mice used in this work followed institutional animal welfare guidelines and legislation, as approved by the local Animal Ethics Committee (Comitato di Bioetica e Valutazione, Torino, Italy, authorization no. 286/2019-PR).

### Data availability.

The data that support the findings of this study are available within the article, the supplemental material, and the Supporting Data Values files or from the corresponding author upon reasonable request. The code used to compute single-cell subcellular distribution is available in GitLab (https://gitlab.com/jpmargaria/subclock). A reporting summary for this article is available in the supplemental materials.

## Author contributions

All authors contributed extensively to the work presented in this manuscript. HL, JPM, PZ, LG, MCDS performed most of the experiments and developed the methodology. DT, SP, and FAT analyzed the human BC samples. MGF and EG generated the biological data from the PDX-derived cells. CZ and MF performed and analyzed mouse histopathology. CCC, ST, HL, and JPM profiled RAB11A activity. LP conducted the zebrafish experiments. GV and GS supervised experiments on ITGB1 trafficking. MLJ and JEB designed the SH3BP5L GEF–deficient mutants. JEB built the AlphaFold3 models. HL, JPM, and EH jointly conceived the original idea for the study. JPM, MM, FG, IF, and EH supervised the project, collected the data, and performed the analysis. EH, and JPM wrote the manuscript. The order of the co–first authors reflects minor differences in contribution that were jointly agreed upon by the authors; all designated co–first authors contributed equally to this work. All authors provided final approval of the version submitted for publication. During the preparation of this work the authors used the ChatGPT 4o tool (licensed to the University of Torino) to improve the quality of their scientific writing. All content was subsequently reviewed and edited by the authors, who take full responsibility for the final version of the manuscript.

## Funding support

- Associazione Italiana per la Ricerca sul Cancro (AIRC) (IG-21875 and IG-28862, to EH; IG-28763, to GS; IG-27013, to MM; IG 23049, to SP; MFAG 2020-ID 24897, to CCC; MFAG 2023-ID 27039, to FG; and MFAG 2021-ID 25736, to IF).
- Progetti di Ricerca di Rilevante Interesse Nazionale (PRIN) (202032AZT3, to EH; P20229WSC9, to EH and SP; P2022X4J8F, to MM).
- Fondazione Ricerca Molinette (to EH).
- Internal grant program of the Italian Institute for Genomic Medicine (to CCC).
- Natural Science and Engineering Research Council (Discovery Grant NSERC-2020-04241, to JEB).
- Fondazione CRT grant 2023 (to MM).
- Italian Ministry of Health with Ricerca Corrente and 5×1000 funds (to SP).
- PNRR M4C2-Investimento 1.4-CN00000041 “Finanziato dall’Unione Europea-NextGenerationEU” (to EH).
- Associazione Italiana per la Ricerca sul Cancro (AIRC) postdoctoral fellowship 28201 (to MCDS).
- IEO-Monzino Foundation fellowship (FIEO) (to FAT).
- AIRC fellowship 22558 and Fondazione Umberto Veronesi fellowship (to JPM).

## Supplementary Material

Supplemental data

Unedited blot and gel images

Supporting data values

## Figures and Tables

**Table 1:**
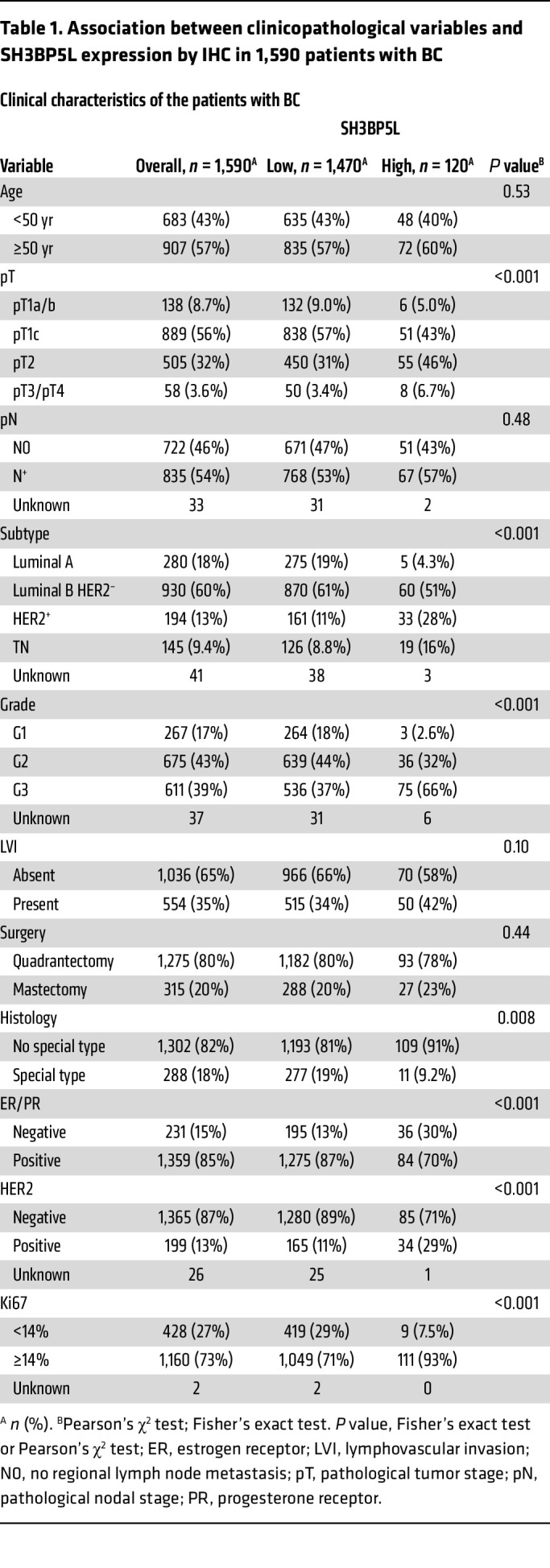
Association between clinicopathological variables and SH3BP5L expression by IHC in 1,590 patients with BC

## Version 1 02/02/2026

Electronic publication
