# Supplementary material for: SH3BP5L triggers the RAB11A-regulated integrin recycling network implicated in breast cancer metastasis
Source: J Clin Invest. 2026 Feb 2;136(3):e192705. doi: 10.1172/JCI192705 (PMC12867135; doi:10.1172/JCI192705)
Supplement: Supplemental data [file jci-136-192705-s312.pdf]

SH3BP5L triggers RAB11A-regulated integrin recycling network implicated in breast cancer metastasis

Huayi Li[1,*], Maria Chiara De Santis[1,*], Francesco A. Tucci[2,3,*], Daniela Tosoni[2,3,*], Ping Zhang[1,*], Meredith L. Jenkins[4], Giulia Villari[5,6], Maria Grazia Filippone[2], Elisa Guerrera[2,3], Simone Tealdi[6,7], Luca Gozzelino[1,6,8], Federico Gulluni[1], Lorenzo Prever[1], Cristina Zanini[1], Marco Forni[1], Irene Franco[9], Miriam Martini[1], John E. Burke[4,10], Guido Serini[5,6], Carlo Cosimo Campa[6,8,#], Salvatore Pece[2,3,#], Jean Piero Margaria[1,11,#,§], Emilio Hirsch[1,#,§].

1. Department of Molecular Biotechnology and Health Sciences. Molecular Biotechnology Center, University of Torino, Torino, 10126, Italy.
2. IEO, European Institute of Oncology IRCCS, Milan, 20139, Italy
3. Department of Oncology and Hemato-Oncology, University of Milano, Milano, 20142, Italy
4. Department of Biochemistry and Microbiology, University of Victoria, Victoria, British Columbia, V8W 2Y2, Canada.
5. Department of Oncology, University of Torino Medical School, Candiolo, Torino, 10060, Italy
6. Candiolo Cancer Institute, Fondazione del Piemonte per l'Oncologia (FPO) - IRCCS, Candiolo, Torino, 10060, Italy
7. Department of Mechanical and Aerospace Engineering, Politecnico di Torino, Torino, 10129, Italy
8. Italian Institute for Genomic Medicine, Candiolo, Torino, 10060, Italy.
9. Università Vita-Salute San Raffaele Milan, 20132, Italy
10. Department of Biochemistry and Molecular Biology, The University of British Columbia, Vancouver, British Columbia, V6T 1Z3, Canada.
11. Somatic mutation mechanisms Unit, Division of Genetics and Cellular Biology, Ospedale San Raffaele - IRCCS, Milan, 20132, Italy.

* HL, MCDS, FAT, DT and PZ equally contributed as first authors

\# CCC, SP, JPM and EH equally contributed as co-last authors

§ JPM and EH are co-corresponding authors

Jean Piero Margaria, PhD

Somatic mutation mechanisms Unit

Division of Genetics and Cell Biology

IRCCS San Raffaele Hospital

Via Olgettina 58, 20132 Milan, Italy

Tel. 0039 02 2643 2357; E-mail: margaria.jean@hsr.it

Emilio Hirsch, PhD

Department of Molecular Biotechnology and Health Sciences.

Molecular Biotechnology Center (MBC),

University of Torino, Via Nizza 52, 10126 Torino, Italy

Tel. 0039 011 670 6425; Email: emilio.hirsch@unito.it

Conflict of interest

EH is a founder of Kither Biotech, a company involved in the development of PI3K inhibitors. E.H. and C.C.C. own patents related to quantification of Rab11 activity.

Supplemental Material

Supplemental Methods

Antibodies

The following antibodies were used in this study: mouse-anti-RAB11(BD Biosciences 610656, WB 1:1,000); mouse-anti-FLAG (SIGMA clone M2, WB 1:2,000); rabbit-anti-SH3BP5L (Novus Biologics NBP2-38385, WB 1: 1,000); rabbit-anti-SH3BP5（SIGMA R81104，WB 1:1,000）; rabbit-anti-KIF5B（Proteintech 21632-1-AP, WB 1:1,000）; mouse-anti-Vinculin (gift from Emilia Turco, University of Turin, Italy, WB: 1:10,000); rabbit-anti-mcherry (Cell signaling #43590, WB 1:1,000) CellMask orange (Thermofisher C10045, 1:5,000); rabbit-anti-GAPDH (Cell signaling #2118, WB 1:1,000); mouse-anti-GFP (Cell signaling #28064, WB 1:1,000); rabbit-anti-RCAS1 (Cell signaling #12290, IF 1:100); rabbit-anti-LAMP1 (Cell signaling #9091, IF 1:100); mouse-anti-HA-tag (Invitrogen #26183, WB 1: 1,000); rabbit-anti-RAB11A (Invitrogen #71-5300, WB: 1: 1,000); RAT-anti-active ITGB1 (BD Pharmingen, clone 9EG7, 553715); RAT-anti-inactive ITGB1 (BD Pharmingen, clone MAb 13, 552828); mouse-anti-ITGB1 (Abcam, clone P5D2, ab24693); mouse-anti-Integrin α5 （SCBT sc-19668）; mouse-anti-Integrin α6 （SCBT sc-374057）; mouse-anti-Integrin αV （SCBT sc-9969）; mouse-anti-Integrin α3 （SCBT sc-7019）. The following peroxidase linked secondary antibodies were used: Anti-mouse IgG (ab131368, WB 1:5,000) and Anti-Rabbit IgG (A0545 SIGMA, 1:5,000). The following Alexa-Fluor conjugated secondary antibodies were used: anti-mouse/rabbit IgG Alexa Fluor 488/568/633 (IF 1:1,000).

DNA constructs

SH3BP5-FLAG, SH3BP5L-FLAG, SH3BP5L-GFP, mCherry-SH3BP5L and HA-KIF5B were purchased from VectorBuilder. SH3BP5L$^{(AAA)}$ and SH3BP5L$^{(AK)}$ mutants were generated by site directed mutagenesis (Quikchange Lightning kit, Agilent) using the following pair of primers and verified by sequencing: CTGGTTGATCTCCTCGCTGGCCGCGGCCGCGTGCTCCAACTCCTCCTGTATT and AATACAGGAGGAGTTGGAGCACGCGGCCGCGGCCAGCGAGGAGATCAACCAG for SH3BP5L$^{(AAA)}$ mutant; TGCTCGCTGATCTGCTTCGCGTTACGAAGGGCCACG and CGTGGCCCTTCGTAACGCGAAGCAGATCAGCGAGCA for SH3BP5L$^{(AK)}$ mutant. GFP-KIF5B and pFX-iRFP-RAB11: were purchased from Addgene (Addgene plasmid # 172203; addgene.org/172203; RRID: Addgene_172203 and Addgene plasmid # 174462; addgene.org/ 174462; RRID: Addgene_174462). Purchased plasmids were tested by restriction digestion and sequencing. GST-RAB11FIP3, TBC1D98, RAB11A-GFP-wt, RAB11A-GFP-Q70L, RAB11A-GFP-S25N were made in our previous study (1).

Silencing experiments were performed using the following siRNA for SH3BP5L silencing, SASI_Hs01_00182464 and SASI_Hs01_00182465 siRNAs targeting human mRNA NM_030645 (SH3BP5L_1 5′-CGAGGAAACUGAAUACACA-3′; SH3BP5L_2 5′-CAGGGUUCCUGGCUUGAAU-3) were purchased from Sigma-Aldrich together with SIC001-1NMOL a scrambled siRNA control. For CRISPR/Cas9 mediated knockout, target sequences were designed via a gRNA design tool (Feng Lab CRISPR Design Web Tool at https://portals.broadinstitute.org/gppx/crispick/public). The sequence was cloned into the PX335 plasmid to express the Cas9n (nickase) and a single guide RNAs (sgRNAs) was

transfected in MDA-MB-231 cells to knockout SH3BP5L gene. The following sequence was used for SH3BP5L: 5'- CACCGAGAACCGACTGGACCCCACG-3'. Plasmids and siRNAs were transfected using X-tremeGENE HP DNA Transfection Reagent (Roche Applied Science, Penzberg, Germany) according to the manufacturer's instructions.

Alternatively, silencing experiments were performed using the following custom shRNA lentiviral particles (Vector Builder) for SH3BP5L downregulation: shSH3BP5L_1 (pLV[shRNA]-EGFP:T2A:Puro-U6; Vector ID: VB250411-1418hpg), shSH3BP5L_2 (pLV[shRNA]-EGFP:T2A:Puro-U6; Vector ID: VB250411-1420dea) and Scramble as a control (pLV[shRNA]-EGFP/Puro-U6, Vector ID: VB010000-9526zpu). Target cells were seeded at a density of 50×10^5 cells/well in 6-well plates at the time of transduction. For infection, viral particles were added at a multiplicity of infection (MOI) of 20 or 2 for shSH3BP5L particles or sh Ctrl respectively, in the presence of polybrene (4 µg/ml, Sigma-Aldrich) to enhance transduction efficiency. Cells were incubated with the viral suspension for ~ 8 hr under standard culture conditions (37 °C, 5% CO_2). After incubation, the medium was replaced with fresh complete growth medium. ~ 24 hr later, transduced cells were maintained under puromycin, 2 µg/ml.

Supplemental Figure 1

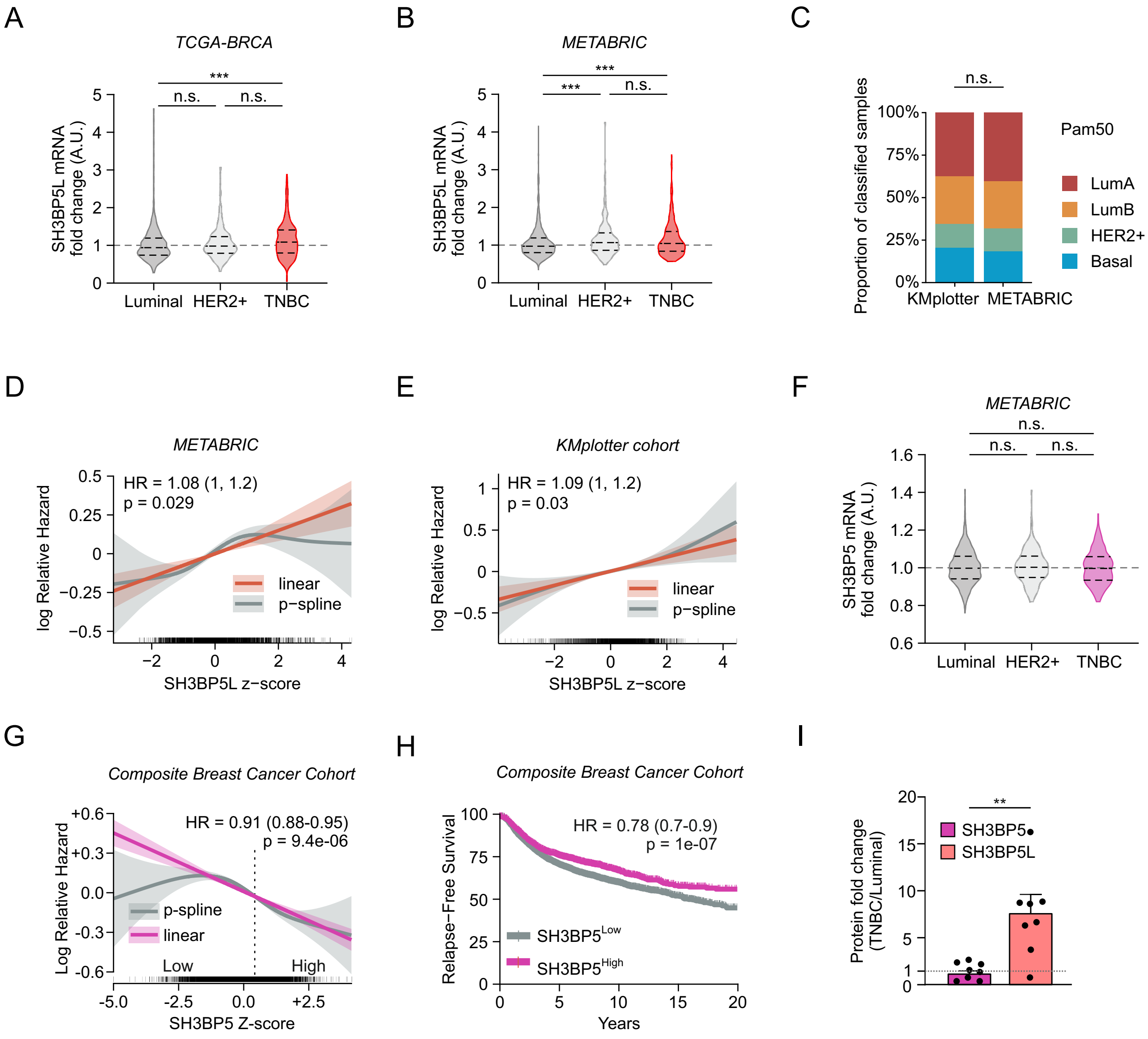

Supplemental Figures

Supplemental Figure 1

Transcriptomic and protein analysis of SH3BP5L and SH3BP5 in breast cancer subtypes. (**A-B**) SH3BP5L mRNA levels in luminal, $HER2^+$ and TNBC breast cancer tumors from the TCGA-BRCA (**A**) and METABRIC (**B**) cohorts. *** $p < 0.001$, Wilcoxon-Mann-Whitney test; n.s.: not significant. (**C**) Bar plot showing the distribution of molecular subtypes according to Pam50 classification in the two cohorts. Normal-like cases were not included in the evaluation, as this subtype is widely interpreted to reflect a quality metric of samples with low tumor cellularity and exceeding contamination of normal tissue rather than an intrinsic subtype. n.s.: not significant by Pearson's chi-squared test. (**D-E**) Continuous analysis of the log relative hazard of RFS against SH3BP5L expression z-scores in the METABRIC and the KMplotter cohorts. Both a linear fit (red) and a penalized smoothing spline fit (grey) are shown. The hazard ratio (HR) and log-rank test p-value refer to the linear term. Shaded areas represent 95% confidence intervals. (**F**) SH3BP5 mRNA levels in luminal, $HER2^+$ and TNBC tumors from the METABRIC dataset. n.s.: not significant, Wilcoxon-Mann-Whitney test. (**G-H**) Association of SH3BP5 expression with relapse-free survival (RFS) in a Composite Breast Cancer Cohort. Continuous analysis of the log relative hazard of RFS against SH3BP5 expression z-scores (**G**). Both a linear fit (purple) and a penalized smoothing spline fit (p-spline, grey) are shown. The hazard ratio (HR) and log-rank test *p*-value refer to the linear term. Shaded areas represent 95% confidence intervals. The dashed line indicates the upper tertile cutoff. Kaplan–Meier analysis of RFS in patients stratified in $SH3BP5^{High}$ (upper tertile) and $SH3BP5^{Low}$ expression (**H**). HR, hazard ratio with 95% confidence intervals; p, log-rank test p-value; n=6902. (**I**) Relative protein quantification of SH3BP5L and SH3BP5 in TNBC compared with luminal breast cancer cell lines, based on Figure 1F. n=8, ** $p < 0.01$, Student t-test.

Supplemental Figure 2

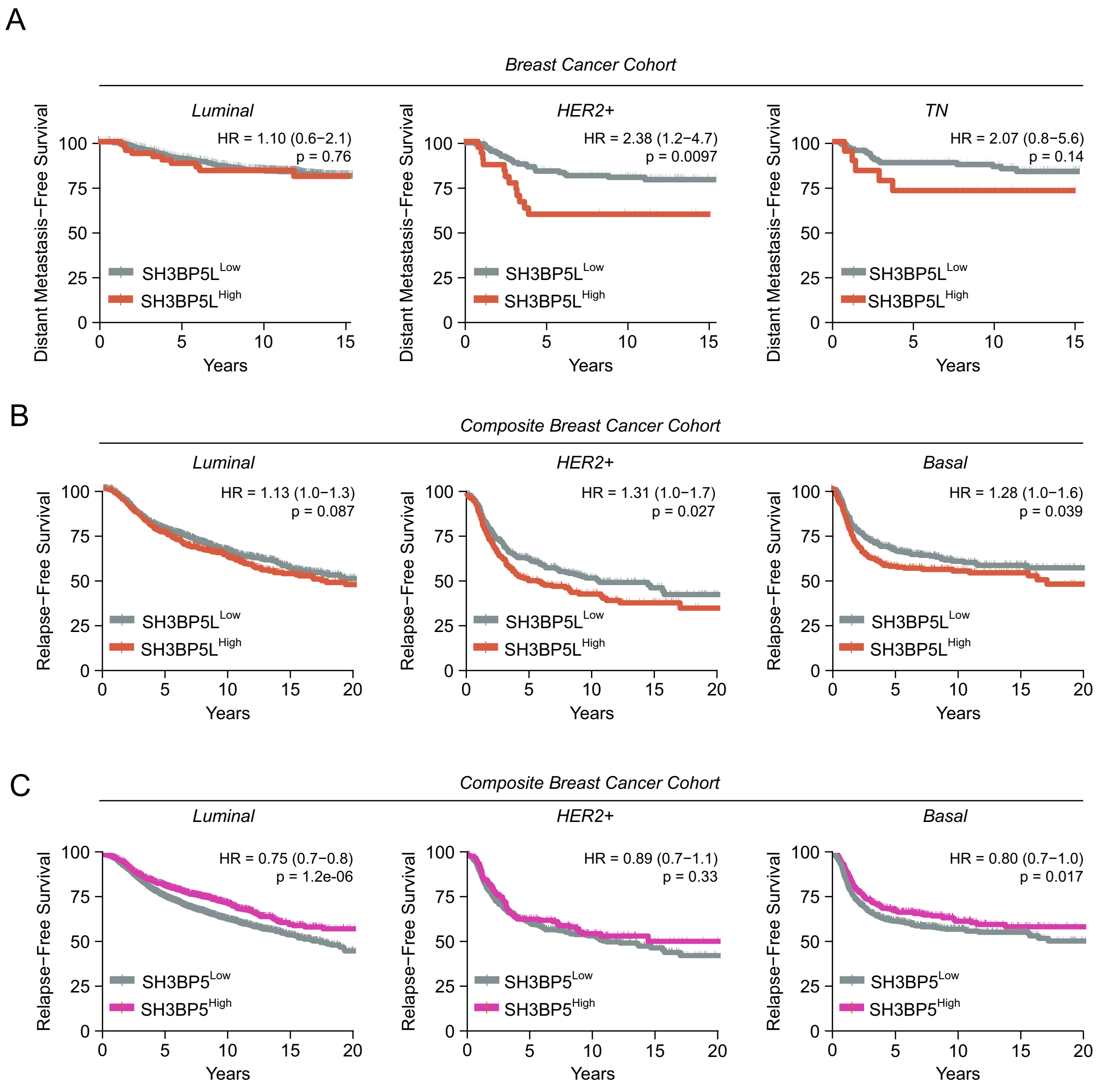

Supplemental Figure 2

Association of SH3BP5L and SH3BP5 expression with prognosis in breast cancer subtypes. (**A**) Kaplan–Meier analysis of distant metastasis-free survival (DMFS) in breast cancer patients stratified by SH3BP5L immunohistochemistry expression (High versus Low) in luminal (n=1210), $HER2^+$ (n=194), and triple-negative breast cancer (TNBC, n=145) subtypes. A significant reduction in DMFS was observed in $HER2^+$ tumors, while no effect was seen in luminal cancers. In TNBC, high SH3BP5L expression showed a similar trend, although the limited number of cases likely restricted statistical significance. HR, hazard ratio with 95% confidence intervals; p, log-rank test p-value. (**B**) Kaplan–Meier analysis of relapse-free survival (RFS) in breast cancer patients stratified by high (upper tertile) versus low SH3BP5L mRNA expression from a Composite Breast Cancer Cohort. HR, hazard ratio with 95% confidence intervals; p, log-rank test p-value in luminal (n=2402), $HER2^+$ (n=598), and basal-like subtypes (n=769). (**C**) Kaplan–Meier analysis of RFS in breast cancer patients stratified by high (upper tertile) versus low SH3BP5 mRNA expression from a Composite Breast Cancer Cohort. HR, hazard ratio with 95% confidence intervals; p, log-rank test p-value in luminal (n=4367), $HER2^+$ (n=935), and basal-like (n=1280) subtypes.

Supplemental Figure 3

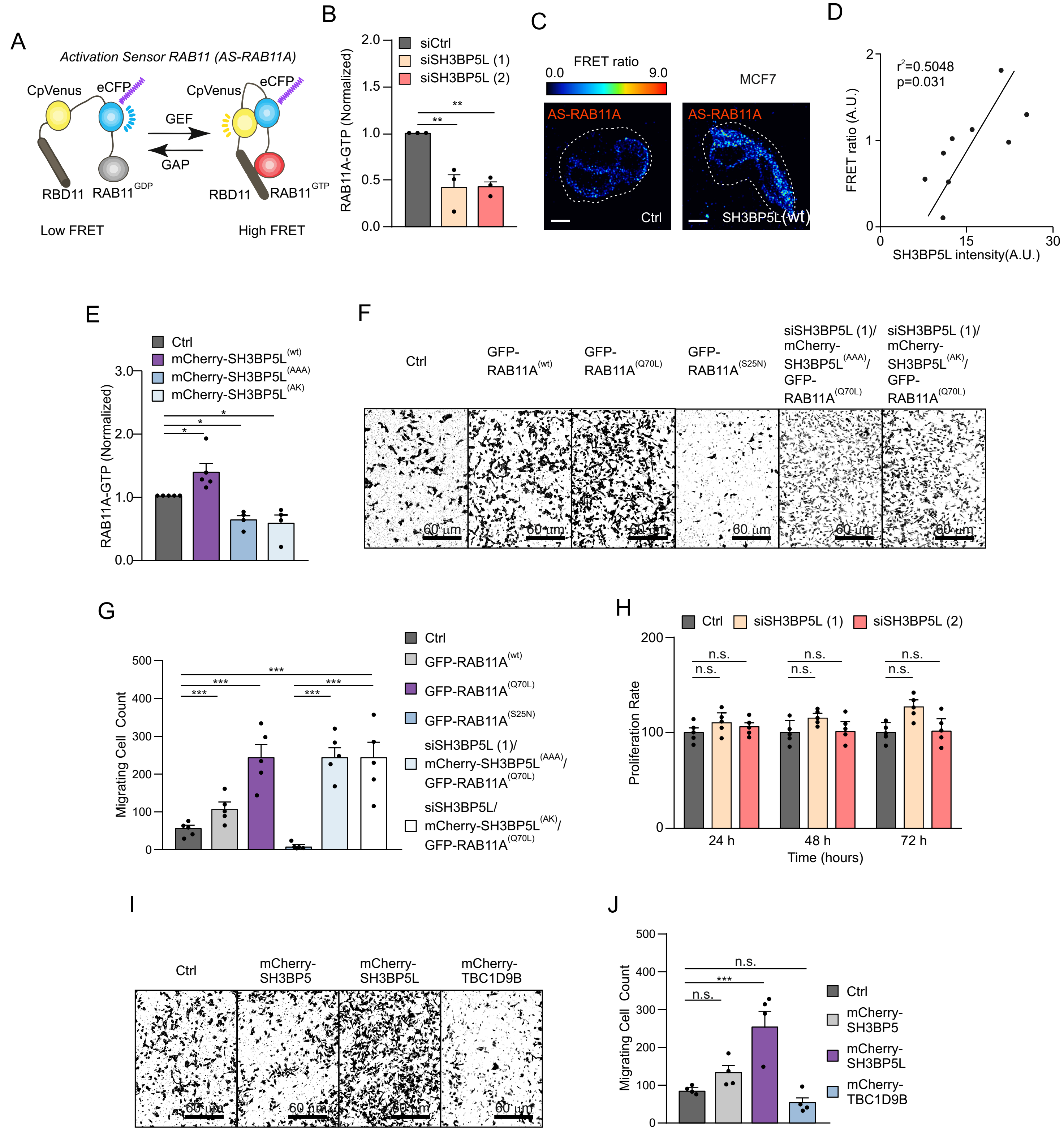

Supplemental Figure 3

SH3BP5L enhances RAB11A-mediated invasion and metastasis. (**A**) Schematic representation showing the activation sensor RAB11A (AS-RAB11A) in low and high FRET status. RBD11: RAB11-binding domain of FIP3 that specifically interacts with RAB11-GTP. (**B**) Relative quantification of endogenous active RAB11 (RAB11A-GTP) shown in Figure 2D. (**C-D**) Representative pseudo-colored confocal image (**C**) and relative quantification (**D**) of FRET ratio in MCF7 cells transfected with AS-RAB11A and SH3BP5L$^{(wt)}$. Scale bar: 5 µm. (**E**) Relative quantification of endogenous active RAB11 (RAB11A-GTP) shown in Figure 2H. (**F-G**) Representative crystal violet staining (**F**) and relative quantification (**G**) of Transwell assay in MDA-MB-231 cells transfected with RAB11A$^{(wt)}$, RAB11A$^{(Q70L)}$, RAB11A$^{(S25N)}$, or MDA-MB-231 cells depleted for SH3BP5L and transfected with constitutively active RAB11A$^{(Q70L)}$, SH3BP5L$^{(AAA)}$ mutant/ SH3BP5L$^{(AK)}$ mutant. Scale bar: 60 µm. (**H**) Proliferation rate of MDA-MB-231 cells transfected with siSH3BP5L (1) and siSH3BP5L (2) at different time points. (**I-J**) Representative crystal violet staining (**I**) and relative quantification (**J**) of Transwell assay in MDA-MB-231 cells transfected with mCherry-SH3BP5, mCherry-SH3BP5L and mCherry-TBC1D9B. Scale bar: 60 µm.

Data represent mean of at least three independent experiments ± SEM, *** p< 0.005, * p< 0.05, n.s.= not significant, 1-way or 2-way ANOVA followed by Bonferroni post-hoc test, where appropriate).

Supplemental Figure 4

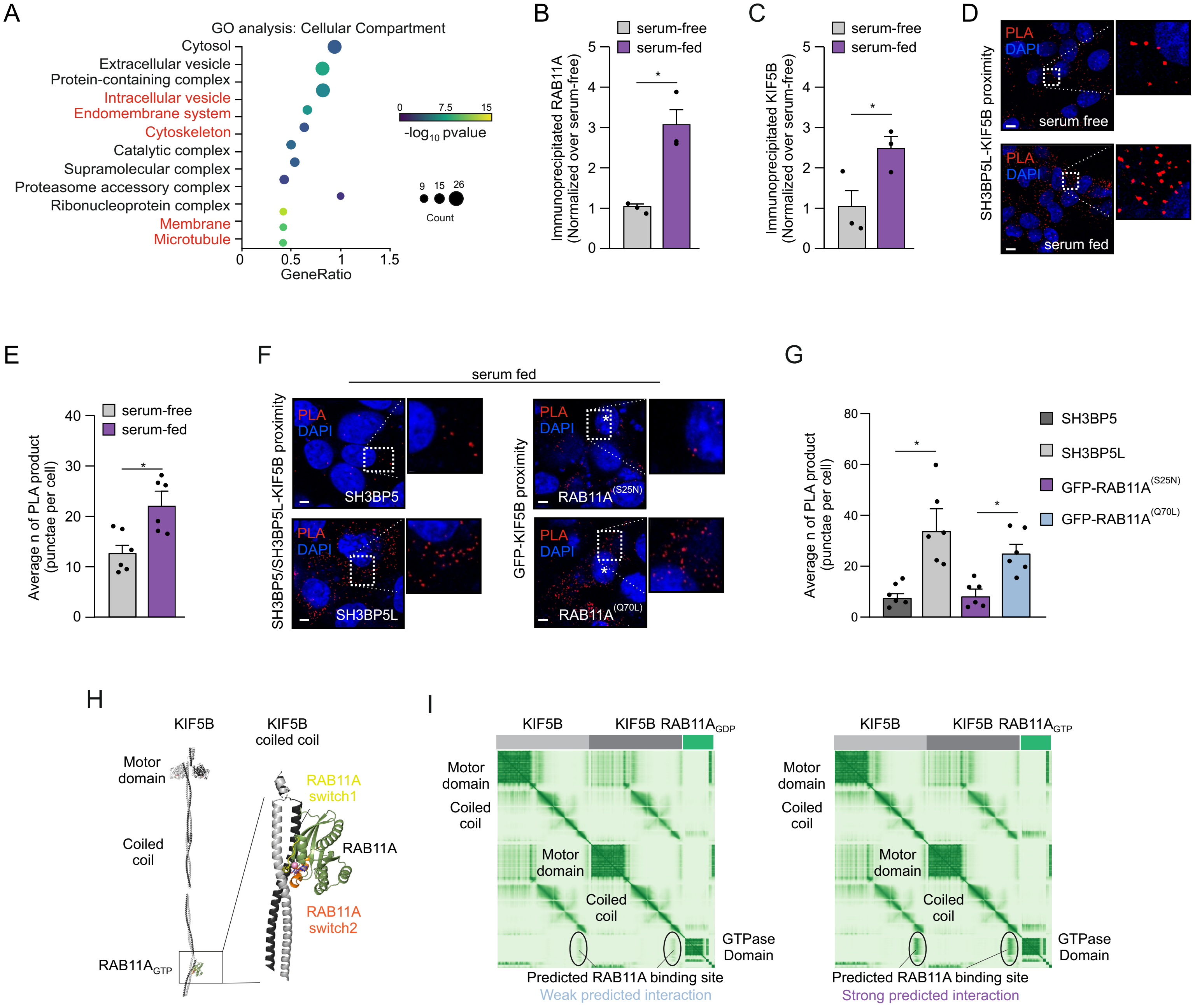

Supplemental Figure 4

SH3BP5L-activated Rab11 promotes KIF5B recruitment. (**A**) Cellular compartment gene ontology (GO) analysis performed on the list of SH3BP5L interactors found by mass spectrometry. (**B**) Relative quantification of RAB11A protein amount in immunoprecipitation performed in serum-free and serum-fed conditions reported in Figure 3D. (**C**) Relative quantification of KIF5B protein amount in immunoprecipitation performed in serum-free and serum-fed conditions reported in Figure 3D. (**D-E**) Representative confocal images (**D**) and quantification (**E**) of PLA assay for SH3BP5L and KIF5B in MDA-MB-231 cells. n = 6 images for each group from three independent experiments, scale bar = 5 µm. (**F-G**) Representative confocal images (**F**) and quantification (**G**) of PLA assay for SH3BP5-KIF5B, SH3BP5L-KIF5B, KIF5B-GFP-RAB11A$^{(S25N)}$ and KIF5B-GFP-RAB11A$^{(Q70L)}$ in MDA-MB-231 cells. Asterisk indicates the positive GFP cells, n = 6 images for each group from three independent experiments, scale bar = 5 µm. (**H**) Alphafold3 modelling of a dimer of KIF5B bound to a single copy of GTP-loaded RAB11A. Search was carried out with 2xATP, which bound to the motor domains of KIF5B. The C-terminal end of the coiled coil (residue 690 onwards) of KIF5B was translated relative to the N-terminal end for clarity, as there was very limited prediction of coil interactions. (**I**) Predicted alignment error for the Alphafold3 search for both GDP and GTP loaded RAB11A bound to a dimer of KIF5B. The predicted RAB11A interaction site is highlighted.

Data represent mean of at least three independent experiments ± SEM, *$p< 0.05$, 1-way ANOVA.

Supplemental Figure 5

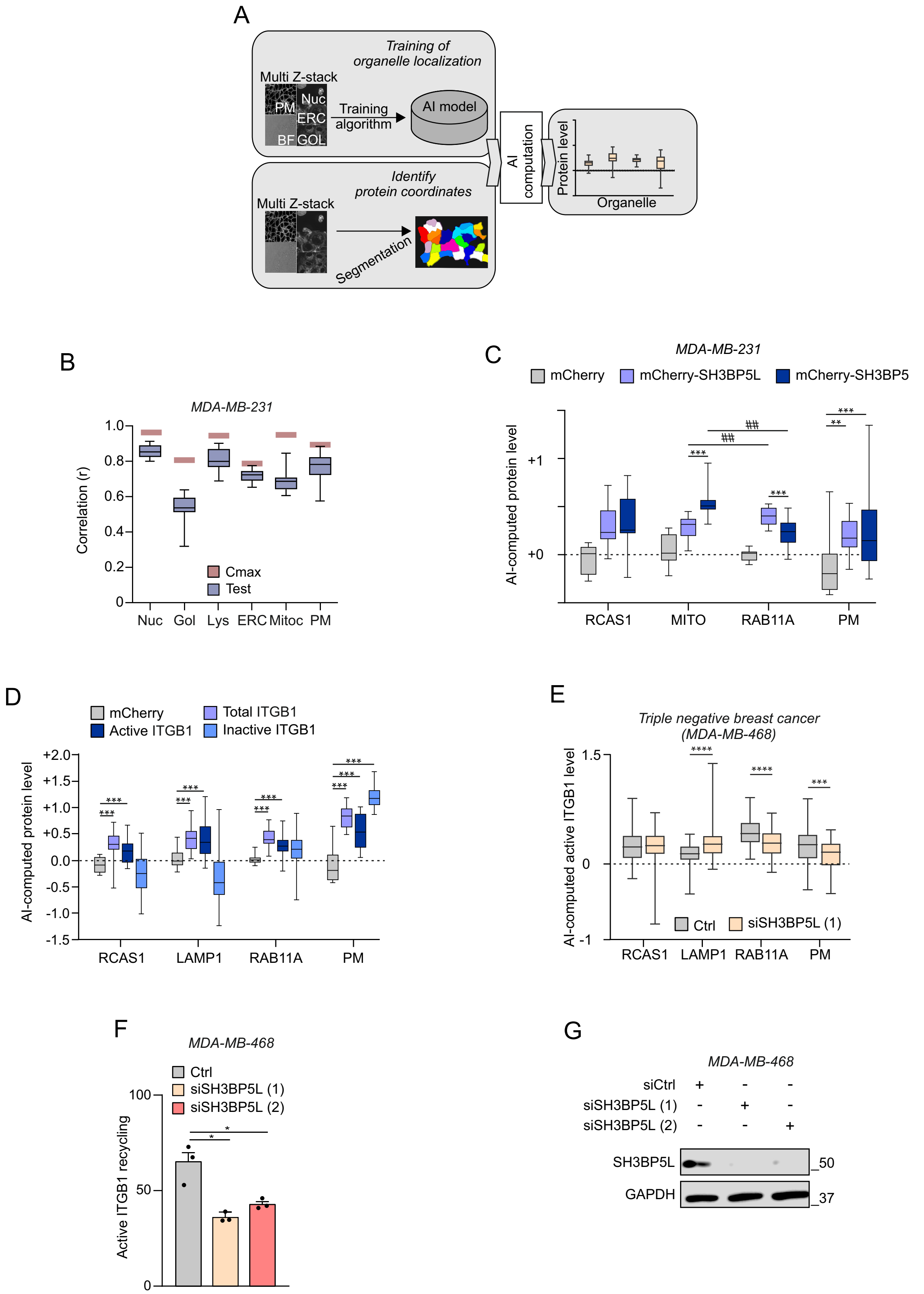

Supplemental Figure 5

SH3BP5L expression modulates ITGB1 localization. (**A**) Schematic representation of immunofluorescence AI-driven analysis of subcellular signals. (**B**) Distributions of the image-wise Pearson correlation coefficient (r) used to evaluate the label-free HCI models. Cmax indicates the average correlation between predicted images and their training references and provides a measure of model training performance (details in Methods and previous description (2)). Test indicates the correlation between predicted images and independent reference images not used for training. Higher values reflect better agreement between predictions and ground truth. The number of test and train images (n) was 30 and 90, respectively for every compartment distribution. Nuc: nucleus, Gol: Golgi, Lys: lysosomes, ERC: endosomal recycling compartment, Mitoc: mitochondria, PM: plasma membrane. (**C**) AI-predicted subcellular distribution of transfected mCherry, mCherry-SH3BP5L or mCherry-SH3BP5 in different cellular compartments of MDA-MB-231 cells. n=8 (mCherry), n=18 (mCherry-SH3BP5L), n=13 (mCherry-SH3BP5) cells respectively. RCAS1: Golgi marker, MITO: MitoTracker, RAB11A: endosomal recycling compartment, PM: plasma membrane stained with CellMask.CellMask (**D**) AI-predicted subcellular distribution of active ITGB1 (9EG7), total ITGB1 (P5D2), inactive ITGB1 (mAb 13) staining, or transfected mCherry in different cellular compartments of MDA-MB-231 cells. n=10 (mCherry), n=28 (active ITGB1), n=28 (total ITGB1), n=40 (inactive ITGB1) cells, respectively. LAMP1: lysosomal marker. (**E**) AI-predicted subcellular distribution of active ITGB1 staining in MDA-MB-468 transfected with either control siRNA (Ctrl, n=68) or SH3BP5L siRNA (siSH3BP5L, n=70). (**F**) FACS quantification of active ITGB1 recycling at 30 minutes in MDA-MB-468 cells transfected with two different siRNA for SH3BP5L. (**G**) Representative western blot image of MDA-MB-468 cells depleted for SH3BP5L with two different siRNAs.

Data represent mean of at least three independent experiments ± SEM, **** $p < 0.001$, *** $p< 0.005$, ** $p< 0.01$, * $p< 0.05$, ##$p < 0.01$, 1-way or 2-way ANOVA followed by Bonferroni post-hoc test, where appropriate.

Supplemental Figure 6

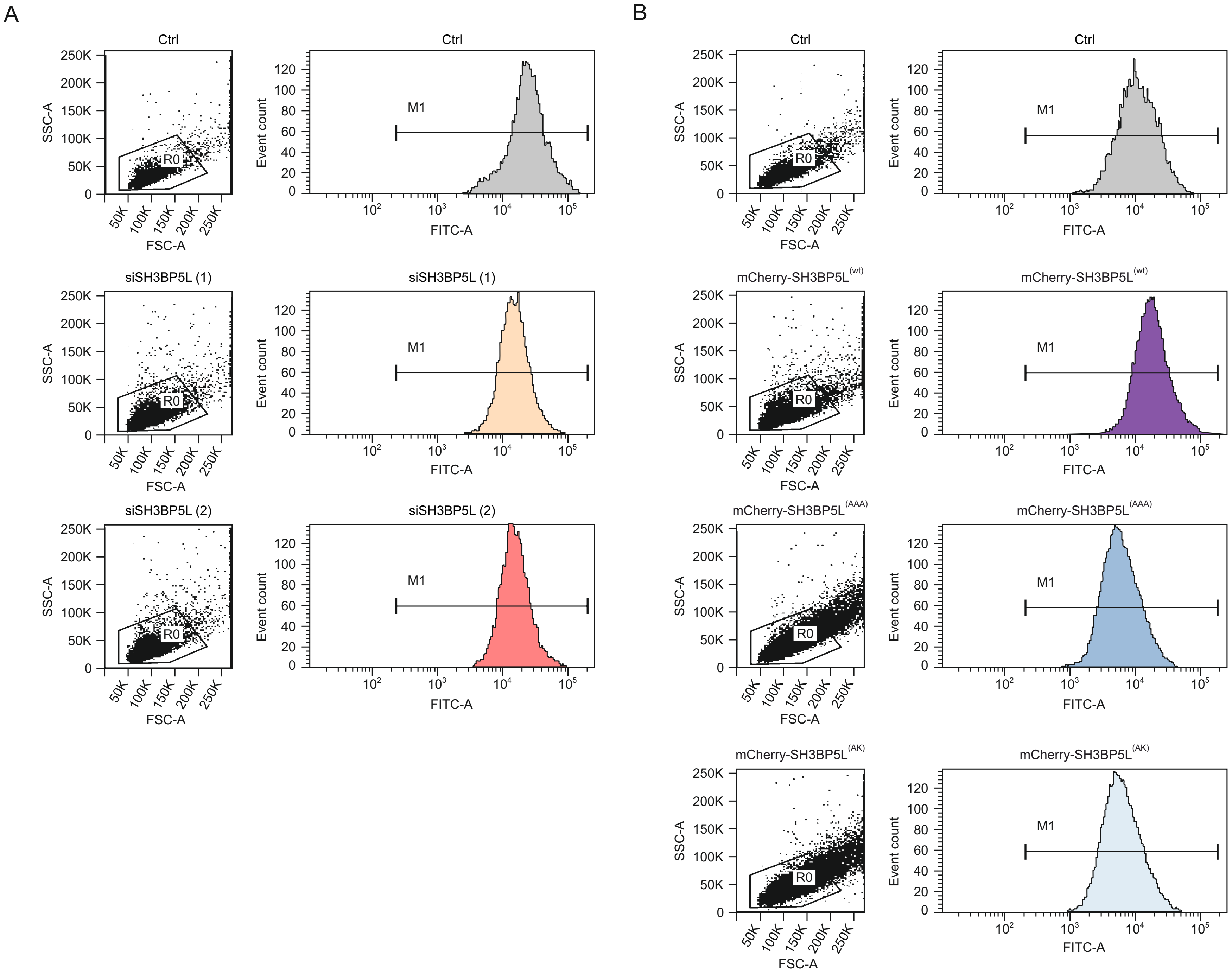

Supplemental Figure 6

Flow cytometry curves. (**A**) Representative flow cytometry curves showing cell-surface re-exposure of active ITGB1 (detected with the conformation-specific 9EG7 antibody, FITC channel) in control MDA-MB-231 cells and in cells transfected with two independent siRNAs targeting SH3BP5L. FSC-A (forward scatter area, proxy for cell size) and SSC-A (side scatter area, proxy for cell complexity/granularity) were used for cell gating, while FITC-A (fluorescein isothiocyanate fluorescence area) reports the antibody signal. A reduction in active ITGB1 recycling is evident following SH3BP5L silencing. (**B**) Representative dot plots and histograms of active ITGB1 recycling in MDA-MB-231 cells expressing mCherry-SH3BP5L constructs. Overexpression of wild-type SH3BP5L [mCherry-SH3BP5L$^{(wt)}$] increased recycling efficiency relative to control, whereas expression of GEF-deficient mutants [mCherry-SH3BP5L$^{(AAA)}$ and mCherry-SH3BP5L$^{(AK)}$] failed to rescue recycling. Data shown correspond to representative experiments of the averaged quantifications presented in Figure 4, F and G.

Supplemental Figure 7

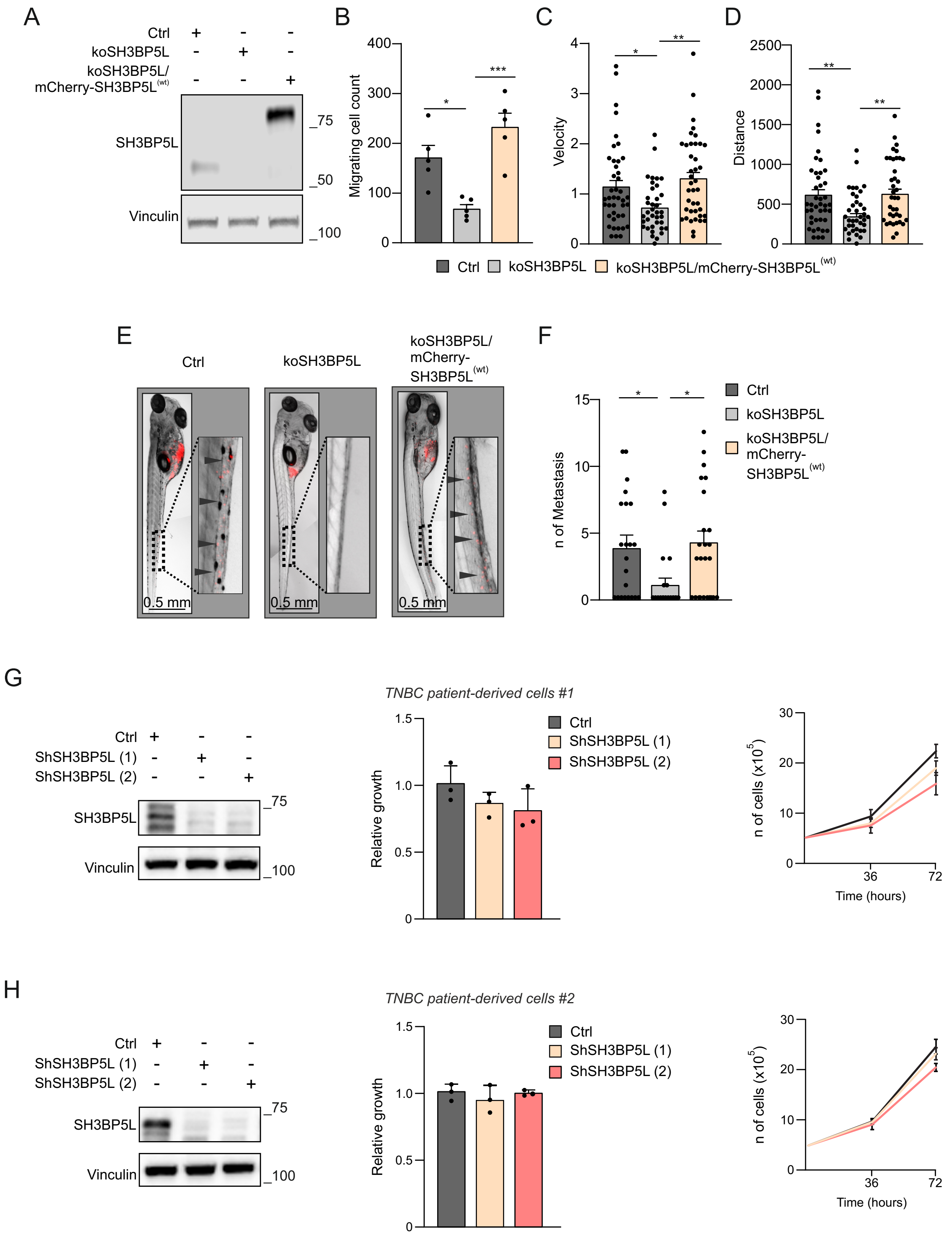

Supplemental Figure 7

SH3BP5L downmodulation reduces invasion and migration of TNBC models. (**A**) Representative western blot image of MDA-MB-231 cells Cas9-mediated knockout for SH3BP5L (koSH3BP5L) and transfected with mCherry-SH3BP5L$^{(wt)}$ construct [koSH3BP5L/SH3BP5L$^{(wt)}$]. (**B-D**) Relative quantification of migrating cells in Transwell assay (**B**), velocity (**C**) and accumulated distance (**D**) in single cell tracking assay, based on Figure 5A. (**E-F**) Representative picture (**E**) and relative quantification (**F**) of metastasized foci in zebrafish injected with MDA-MB-468 cells Cas9-mediated knockout for SH3BP5L (koSH3BP5L) and transfected with mCherry-SH3BP5L$^{(wt)}$ construct [koSH3BP5L/SH3BP5L$^{(wt)}$]. Arrowheads indicate metastatic foci in zebrafish tail. Each dot in the graph (**F**) is representative of an injected zebrafish ($n \geq 20$). (**G–H**) Representative western blot analysis of SH3BP5L expression (left), relative growth measured at 36 hours (middle) and proliferation assay (right) in tumor cells derived from two independent TNBC PDX models. TNBC patient-derived cells #1 (**G**) and #2 (**H**) were transduced with either control or two distinct lentiviral shRNAs targeting SH3BP5L. Data are presented as mean ± SEM (**B-F**) or mean ± SD (**G-H**) of at least three independent experiments, *** $p< 0.005$, ** $p< 0.01$, * $p< 0.05$, 1-way (**B-H** left) or 2-way ANOVA followed by Bonferroni post-hoc test (**G-H** right).

Supplemental Figure 8

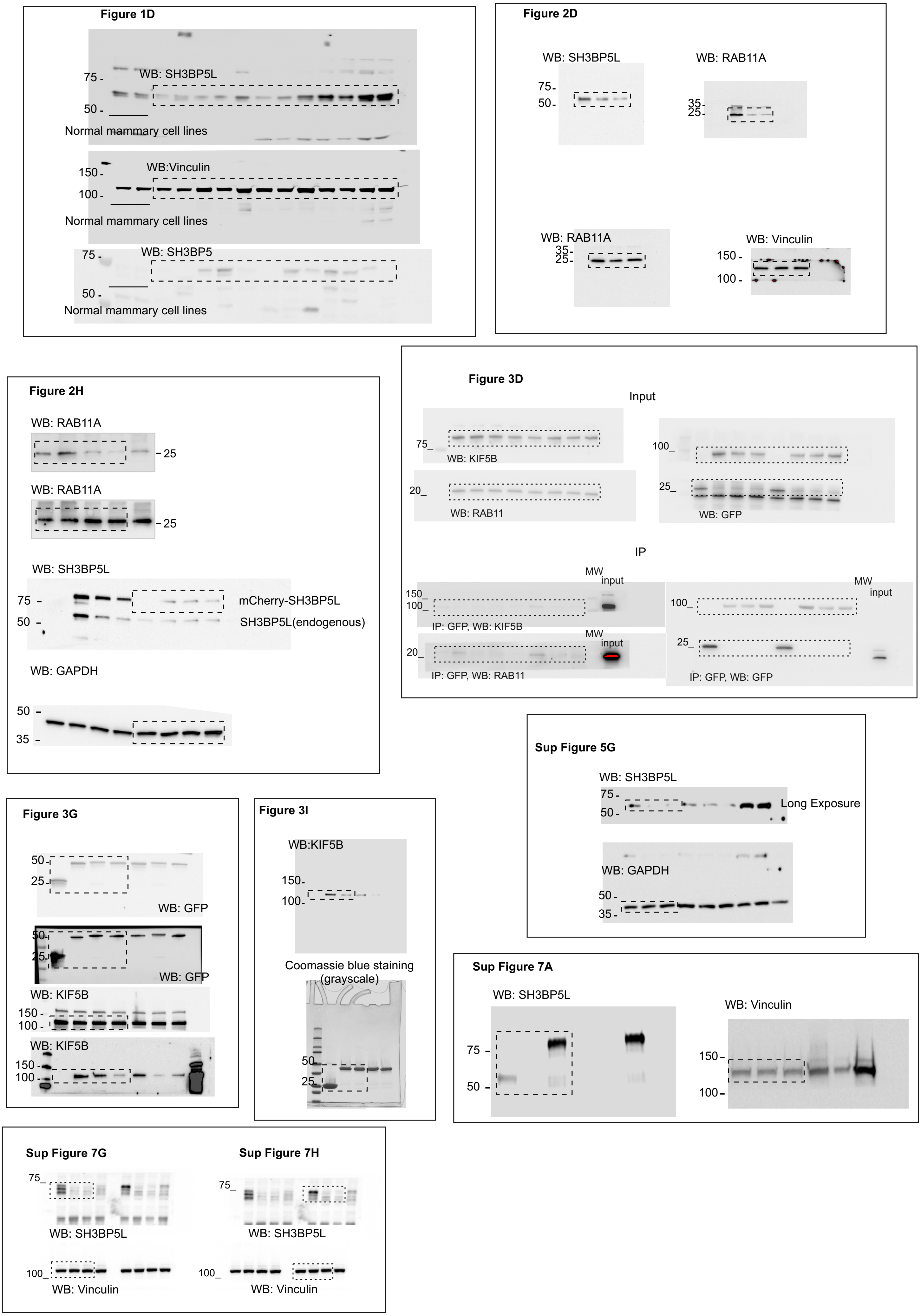

Supplemental Figure 8

Uncropped western blot images. Dotted black squares highlight the cropped part used in corresponding figures.

Supplemental References

1. Campa CC, Margaria JP, Derle A, Del Giudice M, De Santis MC, Gozzelino L, et al. Rab11 activity and PtdIns(3)P turnover removes recycling cargo from endosomes. *Nat Chem Biol.* 2018;14(8):801-10.
2. Ounkomol C, Seshamani S, Maleckar MM, Collman F, and Johnson GR. Label-free prediction of three-dimensional fluorescence images from transmitted-light microscopy. *Nat Methods.* 2018;15(11):917-20.
