# Supplementary material for: SH3BP5L triggers the RAB11A-regulated integrin recycling network implicated in breast cancer metastasis
Source: J Clin Invest. 2026 Feb 2;136(3):e192705. doi: 10.1172/JCI192705 (PMC12867135; doi:10.1172/JCI192705)
Supplement: Unedited blot and gel images [file jci-136-192705-s313.pdf]

Supplemental Figure 8

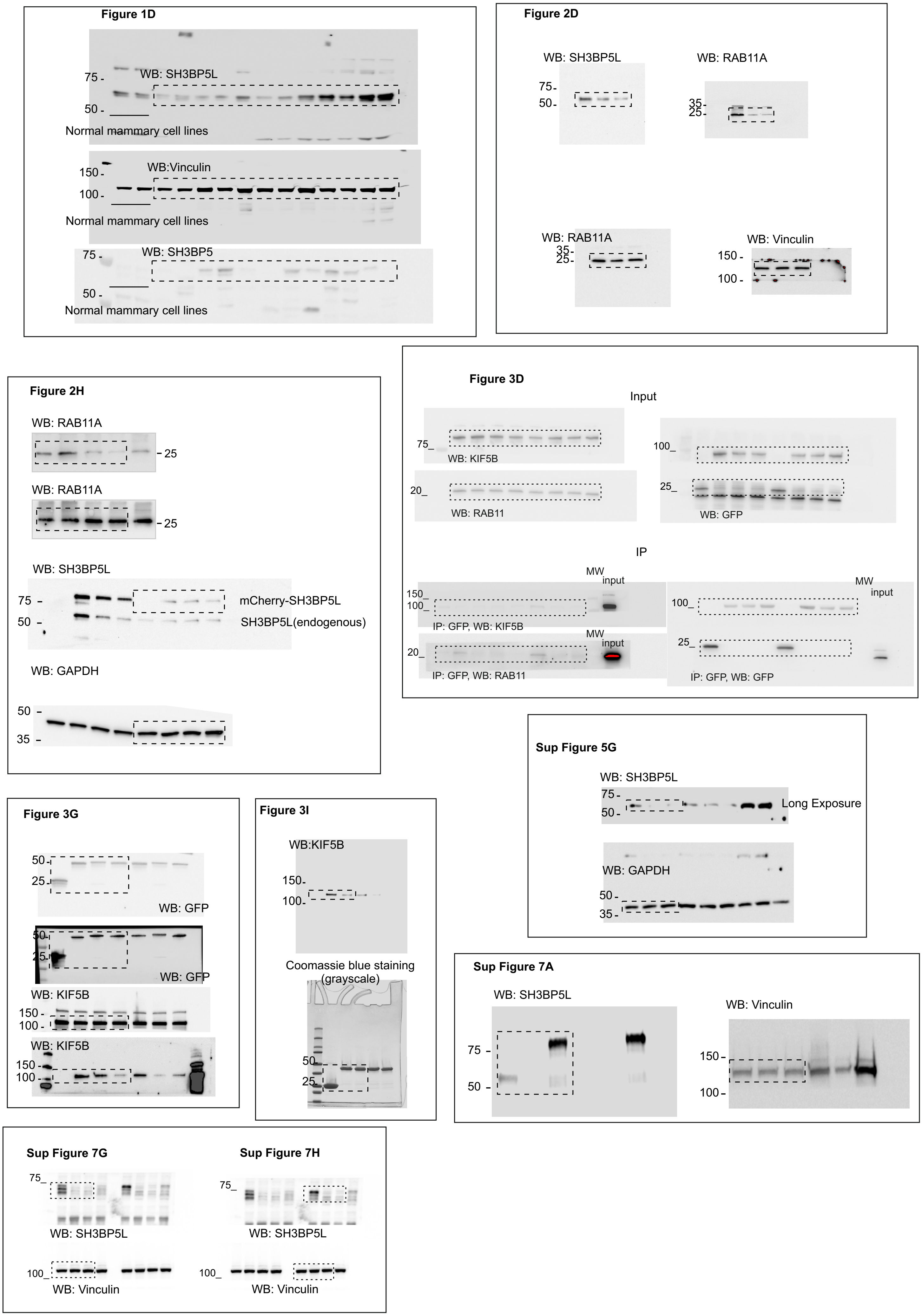

Supplemental Figure 8

Uncropped western blot images. Dotted black squares highlight the cropped part used in corresponding figures.
